# Analysis of transitions at two-fold redundant sites in mammalian genomes. Transition redundant approach-to-equilibrium (TREx) distance metrics

**DOI:** 10.1186/1471-2148-6-25

**Published:** 2006-03-20

**Authors:** Tang Li, Stephen G Chamberlin, M Daniel Caraco, David A Liberles, Eric A Gaucher, Steven A Benner

**Affiliations:** 1 Foundation for Applied Molecular Evolution, Gainesville FL 32604, USA; 2 Department of Molecular Biology, University of Wyoming, Laramie, WY 82071, USA

## Abstract

**Background:**

The exchange of nucleotides at synonymous sites in a gene encoding a protein is believed to have little impact on the fitness of a host organism. This should be especially true for synonymous transitions, where a pyrimidine nucleotide is replaced by another pyrimidine, or a purine is replaced by another purine. This suggests that transition redundant exchange (TREx) processes at the third position of conserved two-fold codon systems might offer the best approximation for a neutral molecular clock, serving to examine, within coding regions, theories that require neutrality, determine whether transition rate constants differ within genes in a single lineage, and correlate dates of events recorded in genomes with dates in the geological and paleontological records. To date, TREx analysis of the yeast genome has recognized correlated duplications that established a new metabolic strategies in fungi, and supported analyses of functional change in aromatases in pigs. TREx dating has limitations, however. Multiple transitions at synonymous sites may cause equilibration and loss of information. Further, to be useful to correlate events in the genomic record, different genes within a genome must suffer transitions at similar rates.

**Results:**

A formalism to analyze divergence at two fold redundant codon systems is presented. This formalism exploits two-state approach-to-equilibrium kinetics from chemistry. This formalism captures, in a single equation, the possibility of multiple substitutions at individual sites, avoiding any need to "correct" for these. The formalism also connects specific rate constants for transitions to specific approximations in an underlying evolutionary model, including assumptions that transition rate constants are invariant at different sites, in different genes, in different lineages, and at different times. Therefore, the formalism supports analyses that evaluate these approximations.

Transitions at synonymous sites within two-fold redundant coding systems were examined in the mouse, rat, and human genomes. The key metric (*f*_2_), the fraction of those sites that holds the same nucleotide, was measured for putative ortholog pairs. A transition redundant exchange (TREx) distance was calculated from *f*_2 _for these pairs. Pyrimidine-pyrimidine transitions at these sites occur approximately 14% faster than purine-purine transitions in various lineages. Transition rate constants were similar in different genes within the same lineages; within a set of orthologs, the *f*_2 _distribution is only modest overdispersed. No correlation between disparity and overdispersion is observed. In rodents, evidence was found for greater conservation of TREx sites in genes on the X chromosome, accounting for a small part of the overdispersion, however.

**Conclusion:**

The TREx metric is useful to analyze the history of transition rate constants within these mammals over the past 100 million years. The TREx metric estimates the extent to which silent nucleotide substitutions accumulate in different genes, on different chromosomes, with different compositions, in different lineages, and at different times.

## Background

Estimation of rate constants for nucleotide substitutions at silent sites of encoding DNA is important to understanding the dynamics of molecular sequence evolution [1-6]. Synonymous substitution can be used draw inferences about functional change in protein, explore the influence of generation time on the rate of sequence divergence[7,8], measure the underlying rate of mutation in natural lineages [9-12], detect different rates of mutation in different lineages [13,14], understand the impact of GC contact on the underlying rate of mutation [15,16], detect covariation in frequencies of substitution [17], detect regions of a genome that may evolve at different rates [19-23], and correlate rates of change with other aspects of genomics [24]. The dynamics of molecular evolution, in turn, is important for inferring information about the fold of proteins [25] and their associated functional behaviours [25]. This, in turn, is critical to making functional assignments to proteins, understanding how that function might have changed historically [26], and correlating changes in biomolecular behavior with the changing palaeontology and geology of Earth and the cosmos [27].

Much literature has discussed the most appropriate way to estimate the number of synonymous and nonsynonymous substitutions separating two sequences. These are frequently expressed as a ratio to the number of synonymous and nonsynonymous sites (*d*_S _and *d*_N_).

This literature was recently reviewed by Yang and Nielsen [6]. These authors commented in particular on what they called "approximate methods" for determining *d*_S _and *d*_N_. Here, the number of synonymous (S) and non-synonymous (N) sites in the sequences are counted. These include silent sites of different degeneracies, including four fold, three fold, and two fold degeneracies, as well as sites that are synonymous or not depending on events at other sites. Approximate methods then count the numbers of synonymous and nonsynonymous differences between the two sequences. They then apply a "correction" to account for the fact that more than one substitution might have occurred at the sites being counted [6].

Yang and Nielsen [6] criticized several of these procedures by noting that they do not accommodate certain well-known features of DNA sequence evolution, such as unequal transition and transversion rate constants, and unequal codon frequencies. These make the counting of sites and differences challenging. These authors then distinguished between four categories of substitutions: synonymous transitions, nonsynonymous transitions, synonymous transversions, and nonsynonymous transversions. The results that emerged from this analysis have been extremely useful in molecular evolution.

The structure of the genetic code permits a more refined type of analysis. In particular, codons within two fold redundant coding systems are, in the universal code, interconverted by transitions only, by purine-purine transitions for the systems encoding Glu (E), Gln (Q), and Lys (K), and by pyrimidine-pyrimidine transitions for the systems encoding Cys (C), Asp (D), Asn (N), Tyr (Y), Phe (F), and His (H).

**Figure 1:**
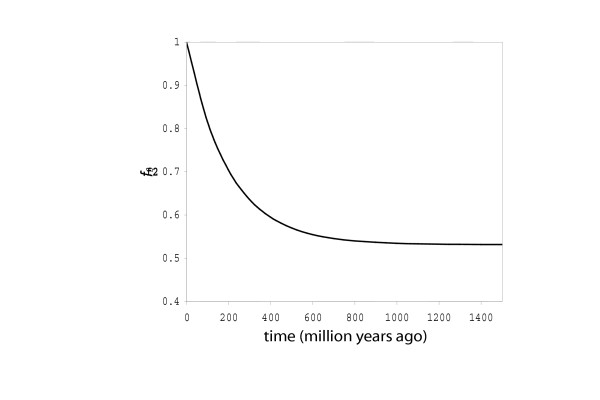
A first order exponential describes the fraction of two fold sites that are identical (*f*_2_) versus the number of changes per site, which can be expressed as process is the consequence Schematic showing the fraction of residues at two fold redundant sites conserved after a time *t*, with an end point of 0.53. Note that in this plot, if we assume that the rate constant for transition is time-invariant, the *x *axis corresponds to time.

For this reason, two fold redundant sites in these systems are expected to follow "approach to equilibrium" kinetics. Such kinetic analysis is well known in chemistry, where it was used by Manfred Eigen to analyze chemical reactions [28]. Given certain assumptions about nucleotide substitution, the fraction of identity at the two fold redundant sites, *f*_2_, is modelled to follow an exponential decay as two sequences diverge, starting at unity and ending at an equilibrium value, typically near 0.5 (but not exactly 0.5) (Fig. 1).

The end point is not exactly 0.5 if the rate constant for the forward transition is not the same as the rate constant for the reverse transition. This difference leads to something that is often mentioned as "codon bias". If the ratio of the rate constants is in the same direction in two lineages whose orthologs are being compared, then the bias will create an end point greater than 0.5. If that ratio is not in the same direction in the two lineages, then the end point will be less than 0.5 (see Methods).

Assuming only that the codon bias is time-invariant, the approach-to-equilibrium kinetic formalism captures in a single exponential equation both the forward and reverse rate processes at two fold redundant sites. This permits us to avoid the "corrections" used in many approaches to capture the possibility of multiple mutations at individual sites. Further, as discussed below, the formalism allows the extraction of specific transition rate constants and equilibrium constants from genomic data, manage directly changing codon biases, and assess the gene-, time-, and lineage invariance of the transition rate constants.

In the past, the TREx formalism has been used to identify pathways in the yeast genome [1] and to analyze the divergence of specific paralogs in mammalian lineages [2]. Here, we apply this formalism to the human, mouse and rat genomes more broadly. An estimate is obtained of the extent to which transitions at two fold redundant sites are invariant in the corresponding lineages, which determines the extent to which a clock based on transition redundant exchanges is overdispersed. We extend this approach in a preliminary way to other vertebrates, to show how it might be used in the future as more vertebrate genomes become available.

## Results

### Calibration of the Transition Redundant Exchange (TREx) dating tool in mammals

**Figure 2:**
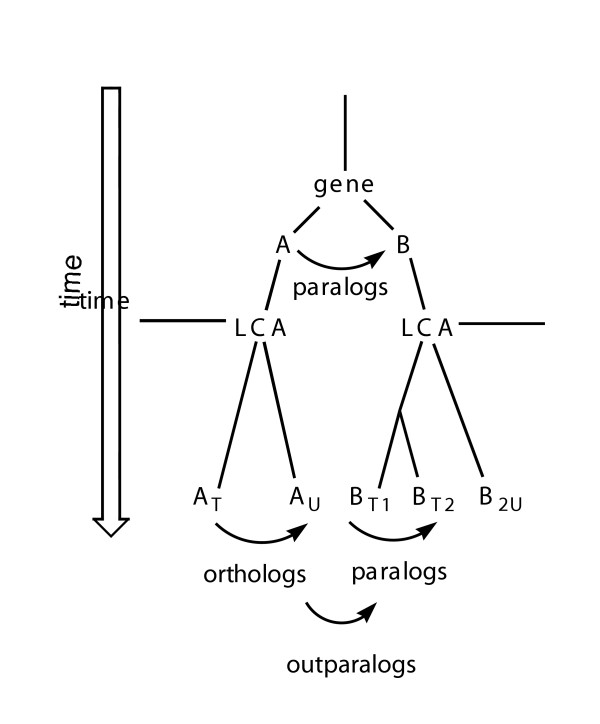
Schematic showing possible intertaxa relationships for a hypothetical gene family that is found in two taxa, *T *and *U*, that shared a last common ancestor (LCA) in which two paralogs of the gene, *A *and *B*, were already present as a consequence of a gene duplication that predates the speciation, after which sequences within lineages *T *and *U *diverged independently. *A*_*T *_and *A*_*U *_represent true orthologs. Pair *B*_*T*1 _and *B*_*T*2 _represent paralogs. Other pairs of modern proteins are neither orthologs nor paralogs.

Immediately after two taxa (*T *and *U*) arise by speciation, each gene in one taxon has a corresponding orthologous gene in the other (Fig. 2). For gene *i*, the two genomes generate the *i*_*T*_:*i*_*U *_pair. Subsequently, individual genes may be lost in separate lineages, removing *i*_*T*_:*i*_*U *_pairs. Genes can undergo further duplication to generate paralogs in one of the two lineages. Such gene duplications subsequent to speciation add intertaxon pairs that, although still often called "orthologs", are associated with different functional implications. It is worth noting that speciation need not be instantaneous, but that the time for speciation is small relative to geological time. Further, as shown below, the time taken to speciate generally falls well within the error of molecular clocks and the fossil record, making it negligible on these time scales as well.

Assuming no lateral transfer, two orthologous proteins in two taxa can have diverged no more recently than the date when the two lineages themselves diverged. Therefore, no clock should date any intertaxon pair as having diverged after two taxa diverged. It is possible, however, for an intertaxon pair to have diverged *before *the two taxa diverged (and be so dated). This will be the case, for example, if the last common ancestor of the two taxa already contained two paralogous genes arising from gene duplication prior to the date of divergence (Fig. 2).

When we consider silent sites within two fold redundant codon systems where the amino acid has been conserved, two fractions measure the extent of the divergence of two sequences. The first, which we denote *f*_2Y_, is the fraction (a number between zero and unity) obtained by dividing the number of sites where the aligned pyrimidines are the same, by the total number of such sites in codons for conserved Cys, Asp, Phe, His, Asn, and Tyr amino acids. The second, which we denote *f*_2R_, is the fraction (also between zero and unity) obtained by dividing the number of sites where the purines aligned are the same, by the total number of such sites in codons for conserved Glu, Gln, and Lys amino acids. Because of these specific constraints, the sites are unambiguously counted.

If all genes in a lineage diverge with the same transition rate constant, then we expect the *f*_2Y _and *f*_2R _values for orthologous pairs to have binomial distributions centered around two means, analogous to the flipping of two coins weighted for the mean values. We can approximate these as normal distributions clustered around midpoint values. These midpoint values will be characteristic of the date when the two species diverged, and the rate constants of pyrimidine-pyrimidine and purine-purine transitions (respectively) in the time since that divergence. Here, "rate constant" is used in the chemical sense, and has the units of changes per site per unit time.

If the replacement of silent nucleotides via these transitions independent at different sites, and if all silent sites in all genes in a lineage behave the same, the breadth of the distribution should depending only on *n*_Y _and *n*_R_, the number of characters used to calculate *f*_2Y _and *f*_2R_, respectively. Thus, if the two genes have relatively few conserved two-fold redundant codons, the distributions of *f*_2Y _and *f*_2R _should be rather large, just as the distribution of the outcome of trials of a coin weighted to come up heads 90% of the time will be broad if the trials each contain only a few coin tosses, but less broad if the trials each contain many tosses.

Other than to the extent expected from a binomial distribution, no pair should have a higher *f*_2Y _or *f*_2R _than the mean characteristic of true orthologs (again, assuming no lateral transfer). In contrast, the *f*_2Y _or *f*_2R _values for outparalogs [29] (Fig. 2), intertaxon pairs that trace their homology through different paralogs present in the last common ancestor, should be smaller than those characteristic of true orthologs (Fig. 2). As the path connecting such pairs can be much longer than the path connecting two true orthologs, their *f*_2Y _and *f*_2R _values can be much lower, even to the point of indicating that the synonymous sites have equilibrated.

**Figure 3:**
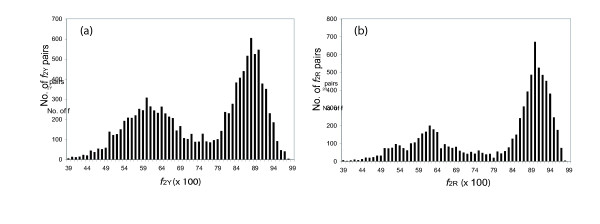
Histogram showing the *f*_2Y _(a) and *f*_2R _(b) values of all mouse:rat intertaxa homolog pairs containing 50 or more characters. The peak centered at ca. 0.88 (a) and ca. 0.90 (b) reflect true orthologs. Pairs with *f*_2 _values near 0.53 diverged so long ago that the silent sites have equilibrated.

Thus, if we compare *f*_2Y _values between homologous pairs of proteins drawn from two species (e.g., mouse and rat), we expect to see a bimodal distribution, with one mode holding pairs having *f*_2Y _values clustering around those expected for true orthologs, the other at much lower *f*_2Y _values. This is in fact seen (Fig. 3). Fig. 3 shows histograms for the *f*_2Y _and *f*_2R _values for mouse:rat intertaxa gene pairs, where the number of sites used to calculate the values (*n*_Y _and *n*_R_) is greater than 50. The histogram shows very few mouse:rat pairs of genes with values of *f*_2Y _or *f*_2R _near unity, a major distribution whose mode is *f*_2Y _= 0.88 and *f*_2R _= 0.90 respectively, and a substantial number of intertaxon pairs that have lower *f*_2Y _or *f*_2R _intertaxa values. Pairs in the distribution centered at *f*_2Y _= 0.88 and *f*_2R _= 0.90 represent presumed rat-mouse orthologs. Pairs having lower *f*_2Y _and *f*_2R _values represent intertaxon comparisons between outparalogs [29].

In the second mode of this bimodal distribution, a substantial number of intertaxon pairs have *f*_2Y _or *f*_2R _values ≈ 0.59. Values of 0.52–0.54 are expected for protein pairs whose silent sites have undergone multiple substitutions, and have therefore equilibrated, if the codon bias is similar in the modern mouse and rat (see below).

The *f*_2Y _and *f*_2R _values of 0.88 and 0.90, with equilibrated end points of 0.51 and 0.54, can be converted to distances based on a simple mathematical transformation, as they are related to distance (changes per site) by an exponential equation (see Methods). These distances (the *k*_obs_*t *values from Methods Equation 20) are additive. For *f*_2Y _and *f*_2R _values of 0.88 and 0.90, TREx distances are calculated to be 0.281 and 0.245. If we assume that the midpoint of the distributions centered at 0.88 and 0.90 correspond to pairs of true orthologs, emerging at the time of the speciation that led to the emergence of independent mouse and rat lineages, and that mouse and rat diverged 16 million years ago [30], this implies that 16 × 2 = 32 million years of total time separate the mouse and the rat. Dividing the observed number of changes per site by the estimated years since divergence, the pyrimidine-pyrimidine and purine-purine transition rate constants can be estimated to be *k*_obsY _= 8.8 × 10^-9 ^changes/site/year and *k*_obsR _= 7.7 × 10^-9 ^changes/site/year (note the units of these rate constants; since generation times are not used to calibrate this clock, no allowance need be made for different generation times in different lineages). It should be noted that the date of divergence of mouse and rat, estimated from the fossil record, is not certain; some molecular clocks estimate the date of divergences to be as old as 40 Ma. The estimates for the rate constants scale linearly with changes in this date of divergence.

### Is the codon bias time-invariant within the mouse-rat clade?

This analysis assumes that the codon bias is time-invariant within this subset of rodents. This is equivalent, under the model, to the statement that the rate constant for a forward transition (for example, the replacement of a T by a C), divided by the rate constant for the reverse transition (in this example, the replacement of a C by a T), is time invariant, even if the rate constants themselves are not. To assess the plausibility of this assumption, we examined the codon bias of mouse and rat. The fraction of T at the two fold redundant sites involving Cys, Asp, Phe, His, Asn, and Tyr (*f*_eqT_) is 0.45 and 0.43 in mouse and rat respectively. The fraction of A at the two fold redundant sites for Glu, Gln, and Lys (*f*_eqA_) is 0.37 and 0.36 in mouse and rat respectively. This suggests that the codon biases have been quite similar in the time separating the divergence of mouse and rat.

From these biases, we calculate expected equilibrium end points for *f*_2R _of 0.53 and 0.54 for mouse and rat respectively, and end points for *f*_2Y _of 0.52 and 0.51 for mouse and rat respectively.

It should be noted that we also assume that the codon bias is equal to the rate constant for the transition of T to C (or, for purines, from A to G) divided by the rate constant for the transition of C to T (or, for purines, from G to A). This is equivalent to the assumption that the codon usage is at equilibrium. This, in turn, is equivalent to saying that the transition rate constant (a first derivative) is larger than the rate of change of the transition rate constant (a second derivative). This is almost certainly the case within closely related mammals; it may not be the case, however, in angiosperm plants, where codon bias seems to be more rapidly changing [34].

### Overdispersion of *f*_2Y _and *f*_2R _values in mouse:rat orthologous pairs

If the transition rate constants in different genes are different (even in the same lineage), then the distribution of *f*_2Y _and *f*_2R _values in orthologous gene pairs will be broader than if the transition rate constants for all gene pairs are the same. We first assumed, as a null hypothesis, that all of the genes represented in the intertaxon pairs have diverged with the same rate constants.

**Figure 4:**
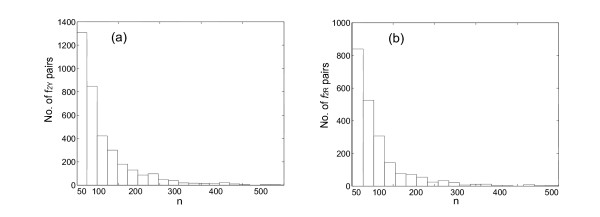
Histogram showing the frequency of *n*, the number of characters used to calculate the *f*_2Y _(a) and *f*_2R _(b) values, in the mouse:rat intertaxa orthologs. The mean (λ) of the Poisson distribution for *f*_2Y _is 136.6 (95% ci 130.1.7–141.2) while the one for *f*_2R _138.2 (95% ci 134.3–140.5). ci: confidence interval. The bin size is 25 sites.

If this is true, then the distribution of *f*_2Y _and *f*_2R _values for orthologs should broader than expected from a binomial distribution. To determine whether these values are "overdispersed", we first calculated the breadth of the expected distribution. As noted above, this depends only on *n*_2Y _and *n*_2*R*_, the number of characters used to calculate *f*_2Y _and *f*_2R_. These numbers are different for different pairs of orthologs. To accommodate this, ca. 3000 mouse:rat pairs having *f*_2Y _and *f*_2R _values distributed around 0.88 and 0.90 (right mode of distributions in *f*_2Y _and *f*_2R_, Fig. 3) were used as mouse:rat orthologous pairs; this was confirmed by phylogenic analysis using the Homologene database (built in May, 2004) and the MasterCatalog (built in March, 2004). The *n*_2Y _and *n*_2R _numbers were then determined for each pair; the distribution of *n*_2Y _and *n*_2R _numbers is shown in Fig. 4. These distributions were fit to a Poisson distribution, and the mean of the distribution (lambda) was calculated.

**Figure 5:**
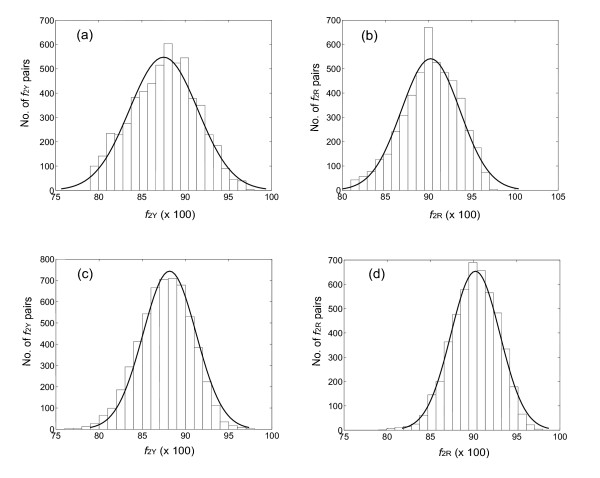
Histograms showing the frequency of *f*_2Y _and *f*_2R _values of mouse:rat intertaxa ortholog pairs. ci = confidence interval. (a). The histogram of observed data (*f*_2Y_) from all ortholog pairs (*n*>50), with the best fit Gaussian superimposed. μ = 0.88 (95% ci 0.877–0.884), σ = 0.040 (95% ci 0.039–0.042). (c). The theoretical histogram from the simulated data that is based on null hypothesis for *f*_2Y _of mouse:rat intertaxa ortholog pairs. μ = 0.88 (95% ci 0.878–0.882), σ = 0.030 (95% ci 0.028–0.031). (b). The histogram of observed data (*f*_2R_) from all ortholog pairs (*n*>50) with the best fit Gaussian superimposed. μ = 0.90 (95% ci 0.880–0.903), σ = 0.034 (95% ci 0.033–0.035). (d). The theoretical histogram from simulated data that is based on null hypothesis for *f*_2R _of mouse:rat intertaxa ortholog pairs. μ = 0.90 (95% ci 0.888–0.903), σ = 0.028 (95% ci 0.027–0.029). ci: confidence interval.

This mean was used as *n*_2Y _and *n*_2R _to calculate the distribution in the *f*_2Y _and *f*_2R _values for intertaxon orthologs that would be expected if all genes diverged with the same rate constant in this lineage (the null hypothesis, see Appendix for details). Gaussian curves were then fit to the observed distributions of *f*_2Y _and *f*_2R _in the intertaxa ortholog pairs (Fig. 5, panels a and c). These distributions had σ values of 0.040 and 0.034, respectively, both modestly larger than the σ values expected from the null hypothesis (0.030 and 0.028, respectively, compare panels a and c, and panels b and d, in Fig. 5). This suggests that the *f*_2Y _and *f*_2R _values are modestly overdispersed. A *χ*^2 ^analysis confirms that the difference between the expected and observed distributions is significant. Thus, we are able to reject the null hypothesis, that all genes in the mouse:rat lineage suffer transitions a TREx sites with the same rate constants.

This observation suggests that at least one of the key assumptions, that the rate constant for transitions is the same at all sites in all genes, is not a perfect approximation to reality. This, in turn, suggests that different ortholog pairs are diverging with different intrinsic rate constants, giving different intrinsic *f*_2Y _and *f*_2R _values for different gene pairs.

The simplicity of the TREx formalism allows a quantitative measure of the extent to which those intrinsic rate constants differ, however. Assuming that the rate constants for different ortholog pairs were distributed log normally, we asked how broad the distribution in intrinsic *f*_2Y _and *f*_2R _values must be to best fit the observed distribution. This required deconvoluting the intrinsic distribution from the distribution arising from a finite value for *n*, and then determining the distribution in the *f *values that might arise from a distribution in the transition rate constants (see Appendix). The σ values associated with the distribution in *f*_2Y _and *f*_2R_values arising from different intrinsic transition rate constants in different genes were ca. 0.019, less than the σ values expected for a simple Gaussian model. This implies that the variation in the rate constants between different genes creates only modest overdispersion in the distribution. In other words, variation in the rate constants for transitions in different genes in the mouse:rat lineage contributes to, but does not dominate, the variance observed in *f*_2Y _and *f*_2R_.

### Aggregating *f*_2Y _and *f*_2R_

One of the shortcomings of the *f*_2Y _and *f*_2R_metrics is that they are each based on only 20–30% of the codons in a pair of genes. A plot of *f*_2Y _versus *f*_2R _(not shown) showed that the *f*_2Y _and *f*_2R _values for the mouse:rat intertaxon pairs were reasonably correlated, and that the pyrimidine-pyrimidine and purine-purine transition exchange rate constants differed by only 14%. As the means of the *f*_2Y _and *f*_2R _distributions are therefore not greatly different, we combined sites undergoing synonymous pyrimidine-pyrimidine and purine-purine transitions to obtain a metric having a smaller sampling error. We asked whether this advantage was associated with a corresponding increase in the dispersion of the metric, which would be expected if the centers of the *f*_2Y _and *f*_2R _distributions were greatly different. This combined metric was termed *f*_2_.

**Figure 6:**
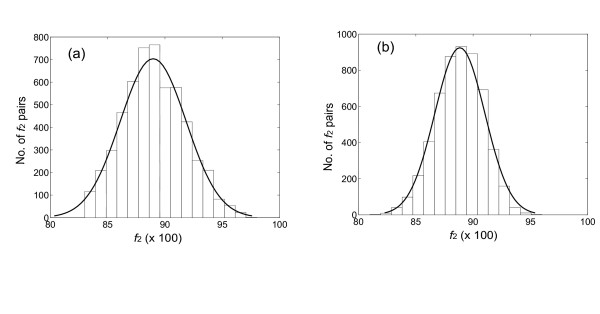
Histogram showing the frequency of *f*_2 _values of mouse:rat intertaxa ortholog pairs. (a). Observed data from all ortholog pairs (*n*>100), with the best fit Gaussian superimposed. μ = 0.89 (95% ci 0.886–0.893), σ = 0.029 (95% ci 0.028–0.031). (b). Theoretical histogram that assumes the null hypothesis that all sites diverge with equal rate constants, based on a simulation with the same distribution of characters. μ = 0.89 (95% ci 0.888–0.891), σ = 0.022 (95% ci 0.021–0.024). ci: confidence interval.

The *f*_2 _histogram for the region for mouse:rat intertaxa orthologous pairs is shown in Fig. 6. The greater number of characters used to calculate *f*_2 _permitted us to examine only those pairs where n>100. Accordingly, the distribution was sharper, with a σ_app _value, which is derived from a Gaussian fit to the experimental data, equal to 0.029. The corresponding simulated data (again assuming all orthologous pairs diverged with the same rate constant, and the mean value for *n*_2 _obtained by a Poisson fit) had a σ value of 0.022. Again, a *χ*^2 ^test showed that the difference was significant, suggesting that there is a difference in the rate constant in different genes as *f*_2Y _and *f*_2R_. The ratio (R_mv_) between σ and μ of *f*_2*R*,_*f*_2Y _and *f*_2 _was calculated (Table 1); the smaller R_app _value, the less variation of the metric. The R_app _value of *f*_2 _is smaller than those of *f*_2R _and *f*_2Y_, demonstrating that *f*_2 _metric is better than the *f*_2R _and *f*_2Y _metrics individually.

**Table 1:** Comparison of *f*_2R_, *f*_2Y_, *f*_2 _and *f*_4_

	μ	σ	R_mv_
*f*_2R_	0.9	0.034	0.0378
*f*_2Y_	0.88	0.040	0.0455
*f*_2_	0.89	0.029	0.0326
*f*_4_	0.84	0.05	0.0595

### Applying the f_2 _metric to the primate-rodent divergence

**Figure 7:**
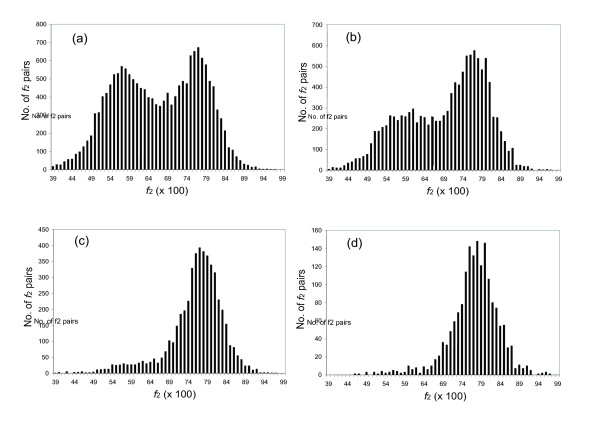
Histogram showing the frequency of *f*_2 _values for intertaxa ortholog pairs (*n*>100) between humans and rodents. (a) Human:mouse ortholog pairs. (b) Human:rat ortholog pairs. (c) Human:mouse ortholog pairs; for pairs from families that had more than one intertaxon pair, the pair with the highest *f*_2 _value is taken, to preferentially extract orthologs. (d) Human:rat ortholog pairs; for pairs from families that had more than one intertaxon pair, the pair with the highest *f*_2 _value is taken.

Moving back in time, the *f*_2 _metric was then applied to examine human:rat and human:mouse intertaxa sequence pairs (Fig. 7). Here, the true orthologs arose from duplications at the time of speciation ca. 10^2 ^million years ago (Ma). Again, the observed distribution in *f*_2 _values was bimodal. The modes of the distribution representing orthologs for the human:rat and human:mouse comparisons are both *f*_2 _= 0.78. The codon biases used in humans are 0.37 and 0.45, respectively for *f*_eqA _and *f*_eqT_, close to those in rodents. Using the human codon bias values, we calculated the expected end points for *f*_2R _of 0.53 and for *f*_2Y _of 0.50 in human. These are similar to those calculated for rodents, suggesting again that the codon bias was essentially invariant in the ancestral organisms separating rodents from primates.

Although the distribution in Fig. 7 is bimodal, the two modes are not as cleanly separated as they are in the mouse:rat comparison. Again, the left mode is interpreted as representing intertaxon pairs that are human:rodent outparalogs, arising because duplications more ancient than the primate:rodent divergence generated paralogs in the last common ancestor of primates and rodents (causing them to have lower *f*_2R _and *f*_2Y _values). This is expected, of course, as the divergence of primates from rodents (ca. 85 Ma) is more ancient than the divergence of mouse from rat.

To use *f*_2 _values to distinguish between orthologous human:rodent pairs and outparalogous human:rodent pairs, we returned to a phylogenetic analysis. A family of proteins that had paralogs in the last common ancestor should give rise to both orthologous and outparalogous pairs from its descendents (Fig. 2). The latter diverged after the former. We therefore expect that within a family, the intertaxon pairs having the highest *f*_2 _values are the true orthologs, while intertaxon pairs having lower *f*_2 _values are outparalogs.

We explored the use of *f*_2 _values to distinguish orthologs from outparalogs within a family for the human:mouse (Fig. 7c) and human:rat (Fig. 7d) intertaxon pairs. Here, for families containing both, only the intertaxon pair with the highest *f*_2 _values was included in the histogram. Obviously, the strategy biases the overall calculation towards slightly higher *f*_2R _and *f*_2Y _values, especially when paralogization occurred in the family just prior to speciation, causing true orthologs and outparalogs to be confused. It fails entirely when the family lacks the true ortholog (either through incomplete gene finding or loss of the true ortholog in one lineage). In Fig. 7c and 7d, the tail towards lower *f*_2R _and *f*_2Y _values presumably reflects gene loss, given that the human and rodent genomes are complete, and gene finding in one included comparison with the others.

The corresponding *kt *values for human:rat and human:mouse orthologous pairs, calculated from *f*_2R _and *f*_2Y _using Eq. 20, the data in Fig. 7c and 7d, and an end point of 0.52, are both 0.613. These are TREx distances. As the time separating human from mouse is the same as the time separating human from rat (the root of the human:mouse:rat tree lies along the segment connecting the node of the tree and the human sequence), the similarity in the TREx distances is expected. From these data, we can conclude that the rate constants for transitions in the lineages represented in the tree by the node-rat and node-mouse branches were the same, for the average gene, within a type I statistics error.

Two options were considered to estimate numerical values for the rate constants for transitions in the time separating human from contemporary rodents. The first assumes that the rate constants were invariant over the entire history. The time *t *since the divergence of rodents and primates is estimated to be ca. 85 Ma [30], making the total time between the two modern species ca. 170 MY. From this, we calculate the average rate constant at two fold redundant sites for the entire episode between modern rodents and human *k*_2 _= *k*_2_*t*/*t *= 3.6 × 10^-9 ^transitions/site/year. This is significantly lower than the rate constant calculated for transitions in the time separating mouse and rat.

It is well known, however, that genes in the mouse:rat lineage evolved more rapidly than genes in the primate lineages [21]. Therefore, an alternative that does not assume time-invariance is preferred. Here, we calculate the rate constant given our knowledge of the rate constants within the mouse:rat lineage. The mode of the *f*_2 _distribution for mouse:rat was 0.89. Assuming an end point of 0.52, this corresponds to a TREx distance of *kt */2 = 0.260/2 = 0.130 from the modern rodents to their last common ancestor, and a rate constant (with a divergence 16 Ma) of 8.1 × 10^-9 ^transitions/site/year (= 0.260/ 32 × 10^6 ^years). As the TREx distances are additive, the TREx distance between the last common ancestor of mouse and rat to human is 0.613 - 0.130 = 0.483. The time from modern humans to the last common ancestor of mouse and rat is 156 MY (86 + 86 -16). This implies that the average rate constant for the period of time separating the ancestor of mouse and rat from humans is 0.483/156 × 10^6 ^years = 3.1 × 10^.-9 ^transitions/site/year. This implies that the transition rate constant at silent sites of two fold redundant codon systems became considerably higher after these rodents diverged.

An analogous analysis can be obtained by explicitly reconstructing the genes in the last common ancestor, and calculating *f*_2 _values from these paired to their human orthologs. Analogous numbers were calculated for other divergences; for example, the transition rate constant within artiodactyls was estimated to be 3.0 × 10^.-9 ^transitions/site/year (data not shown). As no completely sequenced artiodactyls genomes are yet available, this rate constant is based on many fewer data than the rat-mouse-human rate constants.

### Silent two fold sites are not fully equilibrated in time separating mammals and birds

One standard criticism of any distance metric based on silent substitutions is that it cannot be applied to very ancient divergences [1]. As noted above, a clock has maximum accuracy when dating events that occurred one half-life ago. Thus, a clock with a rate constant of 3 × 10^.-9 ^transitions/site/year is maximally accurate when dating events that occurred 116 million years ago (Ma), a time in the mid Cretaceous before the major mammal orders diverged, but after placental mammals diverged from marsupials and monotremes. For those interested in mammalian biology, a clock based on *f*_2 _would appear to be nearly ideal, especially as more genomes are sequenced and individual transition rate constants are calculated for individual branches of an increasingly articulated phylogenetic tree. A clock with this rate constant is, of course, less ideal to study the divergence of vertebrate classes such as birds and mammals, which occurred at ca. 2 half lives ago (ca. 250 Ma).

**Figure 8:**
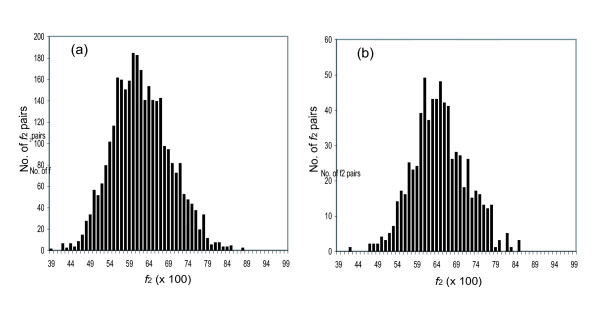
Histogram showing the frequency of *f*_2 _values in chicken:mouse intertaxa gene pairs (*n*>100). (a) All intertaxon pairs. (b) For pairs from families that had more than one intertaxon pair, the pair with the highest *f*_2 _value.

There is no reason to expect, however, that transitions occur at the same rates in mammal lineages in the Jurassic and Cretaceous; such variation is well known, and observed with the TREx metric in different mammalian lineages. We therefore asked whether the TREx metric might be applied to more ancient divergences. A histogram collecting the *f*_2 _values for intertaxon gene pairs from chicken (*Gallus gallus*) and various mammals is shown in Fig. 8. The histogram does not display any obvious bimodality, expected as the orthologous intertaxon pairs are separated by ca. 500 million years. To determine the *f*_2 _value expected for pairs whose synonymous sites were equilibrated, the codon usage in chicken was examined. Codon usage in birds is similar to codon usage in contemporary mammals (*f*_eqA _and *f*_eqT _are 0.38 and 0.42 respectively). If these codon usages are used, then the end points expected for fully equilibrated silent sites are 0.53 and 0.51 for *f*_2R _and *f*_2Y_, respectively. The apparent midpoint of the distribution in the chicken:mammal pairs appears to be higher (ca. 0.63). This analysis suggests (perhaps weakly) that the silent sites used to calculate *f*_2 _have not completely equilibrated in the time separating chickens and humans.

As noted in the discussion of Fig. 7, one way to resolve the overlap between truly orthologous and outparalogous pairs is to include in the histogram only the closest pair of intertaxa proteins within a family, set within a phylogenetic context. The results of applying this strategy to the chicken:mammal pairs is shown in Fig. 8b. As the chicken genome was not complete at the time of this writing, this strategy is expected to be less effective, as many families in the database will not contain the true ortholog from chicken. Nevertheless, the maximum in the histogram shifts to the right (Fig. 8b). This implies that the silent sites are not fully equilibrated in the time separating contemporary birds from contemporary mammals.

**Figure 9:**
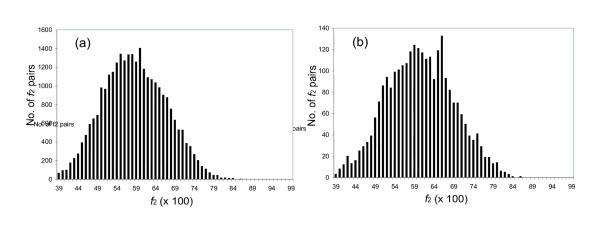
Histogram showing the frequency of *f*_2 _values in tagifugu:human intertaxa gene pairs (*n *>100). (a) All intertaxon pairs. (b) For pairs from families that had more than one intertaxon pair, the pair with the highest *f*_2 _value.

To test the value of this approach where equilibration almost nearly has occurred, we examined the intertaxa distribution for *Takifugu rubipres *(the pufferfish) and human, first where all homolog pairs are recorded (Fig. 9a), and then where only one pair per family is recorded (Fig. 9b). While the differences in the two histograms are not dramatic, and the number of pairs is dramatically reduced, the average *f*_2 _value is shifted slightly to the right in Fig. 9b compared to Fig. 9a. To determine the significance of this shift, we examined the codon bias in the fish genome. The *f*_eqA _and *f*_eqT _are 0.31 and 0.33 respectively, making the expected end point = 0.56. The codon bias is in the same direction as those in other vertebrates, but more exaggerated. This result does not exclude the possibility that silent sites have not fully equilibrated in the time separating contemporary fish from contemporary mammals, but it appears that they have nearly equilibrated. Obviously, as more vertebrate genomes are sequenced, and ancestral genomes more ancient in the lineages of fish and tetrapods are constructed, it may be possible to align ancestral sequences to obtain ancestor-ancestor TREx distances where the equilibration problem is mitigated.

### Comparing *f*_2 _and *f*_4 _metrics

It is possible, of course, to exploit four-fold redundant codon systems at conserved sites to generate an *f*_4 _metric for divergence. All 12 reactions that interconvert the four nucleotides, including both transitions and transversions are silent at 4-fold redundant sites. Further, codon bias in many organisms is more extreme within four fold redundant codon systems, and this codon bias appears to be more likely to change over geological time. Thus, a comparison of the *f*_2 _and *f*_4 _metrics offers an opportunity to determine whether the theoretical advantages proposed for the *f*_2 _metric can be validated experimentally.

**Figure 10:**
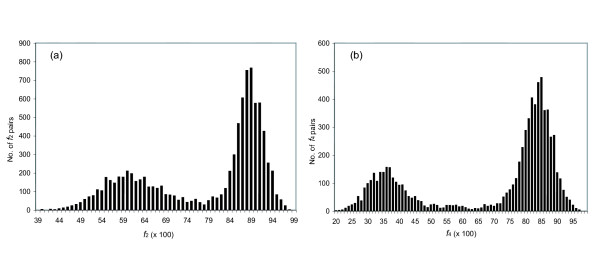
Histogram showing the orthologs of mouse:rat intertaxon pairs using *f*_4 _metric, the fraction identical for four fold redundant codon systems (*n *> 100). While the separation of orthologs from paralogs is larger, the distribution is wider. We do not reject *f*_4 _as a dating tool, but only that its use recognizes its particular advantages (broader sample size) and limitations (greater heterogeneity in microscopic rate constants).

Fig. 10 reports *f*_2 _and *f*_4 _data side-by-side for mouse:rat intertaxon pairs. As expected, the *f*_4 _metric has an equilibrium point that is substantially below the equilibrium point for the *f*_2 _metric. Further, the equilibrium point is not 0.25, which is what would be expected if all four nucleotides were present in equal abundance at equilibrium at the four fold silent sites. Instead, the equilibrium value appears to be somewhere between 0.3 and 0.4, which is consistent with the known codon biases in rodents.

The midpoint of the apparent ortholog distribution in the *f*_4 _histogram for mouse:rat pairs is 0.84, compared to 0.89 for *f*_2_. This is consistent with the lower equilibrium value for *f*_4_, as well as a smaller rate constant for transversions relative to transitions. The R_mv _of *f*_4 _is much greater than that of *f*_2_, suggesting that *f*_4 _clock is more overdispersed than the *f*_2 _clock (Table 1).

### Comparison of the TREx distance with the dS distance analyzing silent substitutions

The maximum likelihood dS metric (mldS), developed by Yang and Nielsen [6] and implemented within the PAML program [31], also analyzes silent substitutions in aligned gene sequences. It has been widely used to describe the evolutionary distance and as a molecular clock [6,32-34]. We therefore compared briefly the features of the TREx and dS metrics.

**Figure 11:**
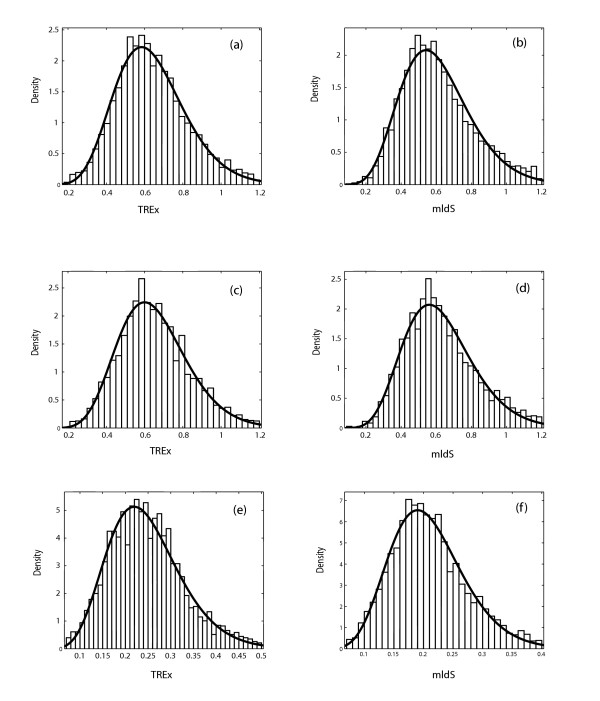
Histogram showing the frequency of orthologs in sister genome pairs, with the best fit Gamma curve superimposed, using TREx and maximum likelihood dS (mldS) metrics: (a) TREx of human:mouse, (b) mldS of human:mouse, (c) TREx of human:rat, (d) mldS of human:rat, (e) TREx of mouse:rat, (f) mldS of mouse:rat.

A set of putative orthologous pairs was extracted from the Homologene database (May, 2004 version). The sequences of each family were aligned using ClustalW and used for the computation of TREx and mldS. The sequence pairs having *n*_2 _values greater than 100 were then extracted, as short sequences cannot be appropriately analyzed using either the TREx or mldS tool. The mldS and TREx distance for each gene pair were calculated and pooled to form a histogram. The outliers in the multigene distributions were trimmed from the dataset and various probability distributions fit to the histograms. The gamma distribution is best fit to all of the histograms of the three genome pairs using TREx and mldS metrics (Fig. 11). R_mv _of TREx is slightly smaller than that of mldS in human:mouse, human:rat while R_mv _of TREx is slightly greater than that of mldS (Table 2). It seems that the TREx metric is better than mldS in the more distant genome pairs, for example, human:mouse and human:rat, while worse than mldS in a closer genome pair, like mouse:rat. However, the differences in these three genome pairs are not significant based on *χ*^2 ^test, indicating that TREx distance is comparable to mldS when estimating distances. TREx is based, of course, on a simpler model and requires much less computation time to calculate than mldS.

**Table 2:** Comparison of the TREx and mldS metrics

	Human:mouse		Human:rat		Mouse:rat	
	TREx	mldS	TREx	mldS	TREx	mldS

μ	0.64	0.61	0.65	0.63	0.25	0.21
σ	0.187	0.202	0.184	0.202	0.081	0.064
R_mv_	0.292	0.332	0.284	0.321	0.325	0.305

## Discussion

Ever since molecular evolution was founded [35], scientists have hoped that some feature of a protein or gene sequence might change at a rate that is sufficiently regular that it could serve as a distance metric. The utility of such a metric is ultimately determined by its ability to support comparisons. At the very least, the metric should be able to compare distances between genes diverging in the same lineage, as these have diverged within the same organismic contexts (e.g, mutation rates and generation times). More ideal would be a clock that would allow the comparison of events recorded in the genome with events recorded in different genomes, or even with events in the geological record [36]. Thee goals generate greater demands, as they require an understanding in lineage-specific difference in rates of divergence, and the connection between sequence divergence and chronological time.

Many features have been considered for this purpose and discarded. For example, the fixation of amino acid replacements does not support well any clock, even for the purpose of comparing divergences of different genes within the same organism [37]. Amino acid replacement are frequently not neutral [38]. In this case, they are driven by purifying selection and/or adaptive evolution, causing episodic (slow or fast) rates of accumulation.

Synonymous nucleotide substitutions in coding regions have been viewed as nearly neutral changes [6,39], which might avoid these problems. Because these substitutions do not change the structure of the encoded protein, they cannot have an impact on fitness at the level of the protein. Indeed, the ratio of nonsynonymous to synonymous substitutions separating a pair of genes is widely used as a metric to detect adaptive evolution in proteins [6]. This metric has been applied to entire genes [40] and entire databases [41], as well as to episodes of evolution represented by branches on evolutionary trees between ancestral sequences [42,43].

The most widely used clocks based on synonymous substitutions aggregate many different types of synonymous substitutions. These include substitutions at two, three, and four fold sites, as well as substitutions at sites that may (or may not) be silent, depending on events at other sites. They also aggregate different chemical processes. At four-fold redundant sites, for example, 12 different rate processes are associated with the conversion of the four standard nucleotides to give each of the three others. There is no reason *a priori *for these rate constants to be similar, let alone identical. Indeed, these are known not to be identical in many lineages. In cases whre they have been examined, transitions are generally faster than transversions [44].

Various scientists have therefore introduced parameters to capture part of this rate variation; the work of Pollock is especially noteworthy [39]. Even with such parameterization, assumptions and approximations remain. Most models assume, for example, that all sites of a kind within a gene accumulate synonymous substitutions with the same rate constants ("site-invariance"), as do all genes within a lineage ("gene-invariance"). It is also frequently assumed that substitution rate constants are the same in all lineages ("lineage-invariance"), and these are the same within a lineage over all epochs ("time-invariance").

Empirical evidence suggests that invariances of these types are only approximations. Evidence for this comes, for example, from the substantial codon biases found at silent sites, biases that can differ greatly between organisms [45,46]. Assuming that the representation of nucleotides at a silent site is in equilibrium in a genome, the ratio of nucleotide Y and X in a genome will be the ratio of the rate constants (having units of reciprocal time) k_*X*->*Y*_/k_*Y*->*X*_, describing the rate of conversion of X into Y and Y into X, respectively. Different codon biases are therefore the consequence of time- and lineage-variant rate constants or, more precisely, variance in their ratios.

This work shows that the silent sites at two fold redundant codon systems are a reasonably useful feature of a coding sequence for supporting distance measurements. Two features of the distributions in the various histograms presented here are noteworthy. The first is their bimodality. The right hand mode is interpreted as representing orthologous pairs, intertaxon pairs of genes that diverged at the same time as the taxa themselves diverged. The left hand mode is interpreted as arising from outparalogs.

This bimodality is expected, rather than a single mode with a long tail towards the left (as expected by one referee). Mammalian genomes contain many families (protein kinase, for example) where multiple paralogs arose prior to the divergence of the principal mammal orders. Many of these arose near the origin of multicellularity. If even modest duplication occurred in a family prior to the divergence of mammals, and if the duplicates have survived, the number of outparalogs in the family will be greater than the number of orthologs. For example, if 4 paralogous families (A, B, C and D) arose before the divergence of mouse and rat, and all of their members survived, the family will generate four pairs of true orthologs (mouseA-ratA, mouseB-ratB, mouseC-ratC, and mouseD-ratD), and add four "counts" to the right hand mode of a histogram. The ancestral duplications will generate six outparalogous pairs. however (mouseA-ratB, mouseA-ratC, mouseA-ratD, mouseB-ratC, mouseB-ratD, and mouseC-ratD), which will contribute to the left mode. The bimodality in the distribution is therefore the consequence of the well-known pattern of recruitment of proteins early in the history of vertebrates. Whole genome duplications are also ways to create outparalogs.

This bimodality also suggests that *f*_2 _values can be used to identify orthologous pairs, especially in incomplete genomes. Here, the *f*_2 _value can be judged as being consistent, or inconsistent, with the hypothesis of orthology, with the measured dispersion in the metric used to assess the likelihood of that judgement. As more tetrapod genomes are sequenced, as the species trees become more highly articulated, as individual branch-specific rate constants are estimated, and ancestral sequences are reconstructed, *f*_2 _values should be generally useful correlating the genomic and geological records.

The second feature of the histograms is that they are modestly more dispersed than expected from a simple binomial distribution. We might ask for the cause of the overdispersion. Kumar and Gadagkar, for example, noted that some of the kinds of non-stationarity in evolutionary processes discussed above might cause clocks to fail. Those that change the composition of sequences at the leaves of the tree can be measured using the Kumar-Gadagkar disparity metric [3].

**Figure 12:**
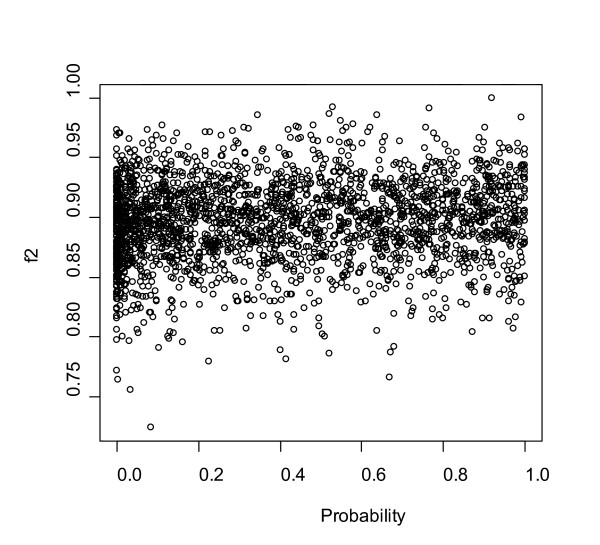
For individual rat-mouse ortholog pairs, a plot of the likelihood that the null hypothesis is rejected under the disparity metric of Kumar and Gadagkar (x axis) versus the *f*_2_. There is no obvious correlation disparity and the *f*_2 _value.

We asked whether the overdispersion in the *f*_2 _metric correlated with the disparity metric. To determine Kumar-Gadagkar disparity, a compositional distance is measured between two sequences, an expected compositional distance is estimated (the null hypothesis), the two are compared, and the probability that the observed distance can be accounted for by the null hypothesis is calculated. Fig. 12 plots this likelihood (x axis) versus *f*_2_. If the outliers in the *f*_2 _distribution arose because the orthologous pairs had a high disparity, a correlation should be observed. One can see a slight trend, whose significance is difficult to evaluate, that is consistent with a correlation between disparity and values of *f*_2 _lower than expected in the distribution.

**Figure 13:**
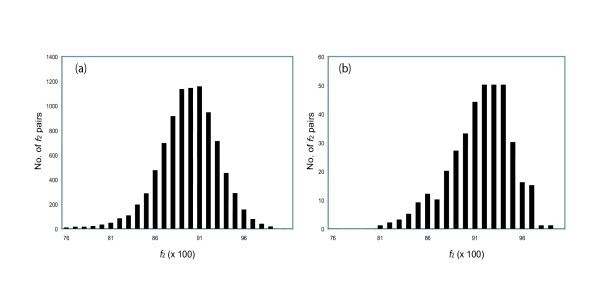
The *f*_2 _values for putative ortholog pairs in rat and mouse are higher if they lie on the X chromosome (panel (b), mean *f*_2 _≈ 0.93) than pairs on autosomal chromosomes (panel (a), mean *f*_2 _≈ 0.90), implying that the X chromosome genes have accumulated fewer silent transitions at two fold redundant sites than the typical pair of orthologs. Since fewer than 5% of the genes lie on the X chromosome, this can account for only some of the overdispersion in the *f*_2 _values for rat-mouse orthologs. Interestingly, an analogous phenomenon was not observed in human-canine ortholog pairs (data not shown).

Another potential source of the overdispersion seen in the histograms is different rates of divergence for different genes on different chromosomes. One suggestion in the literature is that the X chromosome might suffer divergence at different rates from autosomal chromosomes. To explore this possibility, *f*_2 _values were calculated for ortholog pairs in the mouse and rat genomes (where chromosome location is largely conserved), and plotted separately (Fig. 13(b)). Here, it is clear that in the rat-mouse lineage, genes on the X chromosome accumulate transitions at two fold redundant sites more slowly than genes on autosomal chromosome (Fig 13(a)). Separate examination of individual chromosomes (data not shown) revealed that no other chromosome was similarly distinctive in this lineage. Interestingly, although similar behavior was observed in the human-mouse and human-rat comparisons, it was not observed in the human-dog *f*_2 _comparison (data not shown).

In a post-genomic age, with the reconstruction of ancient character states in the sequences of ancient genes and proteins becoming more reliable as each genome is completed, it should be possible to reconstruct the history of these rate constants in specific lineages between specific species. It should be noted that these rate constants will be calibrated from the fossil record, and therefore have the units of reciprocal time. All other things being equal, they are expected (from neutral theory) to be faster in lineages with shorter generation times. In general, calibration for every lineage will be required, as extrapolation of rate constants where the lineage is calibrated to other lineages will not, in general, be justifiable. From lineage-specific calibration, it should be possible to determine how well silent nucleotide substitutions can be modeled as a chemical reaction process over specific lineages through specific epochs. This would support the use of silent substitutions as a molecular clock, if only for the purpose of rejecting character sets that display insufficient invariance to be useful.

To this end, this paper makes four contributions. First, we have described a mathematical formalism, taken from chemical kinetics, which uses rate constants rather than probabilities of transitions to describe substitution at silent sites in encoding genes. This formalism has precedent in the literature of molecular evolution as far back as Jukes and Cantor [47]. It is largely displaced in molecular evolution, however, by a formalism based on statistical language, including reversed Markov Chain models, and the "approximate methods" discussed by Yang and Nielsen [6].

The language avoids the need to "correct" for multiple changes at individual sites. These are frequently required for various transition probability models [6]. When enough corrections are made, the two models converge to the same result. But the mathematical simplicity of the kinetic model will be valuable for a general analysis, and especially an analysis to identify and quantitate changing rates. This includes analyses that calculate rate constants for ancestral lineages between ancestral nodes in an evolutionary tree and the genome-scale comparison.

Second, using this formalism, we have shown for three mammalian lineages (human, mouse and rat) that the variance in the rates for divergence in different gene triplets is not large enough to greatly overdisperse the *f*_2 _metric and can be fit by a model that assumes a modest deviation from a model of gene-invariant transition rate constants.

Third, we have shown in these mammals that a clock based on rate constants for transitions at two fold redundant silent sites is most accurate for divergences occurring ca. 116 million years ago. This makes it extremely valuable to support the analysis of the emergence of new biological function in mammals. An example of this was recently shown for the aromatase gene family in artiodactyls [2]. This was also shown in analyzing paralogs in the yeast *Saccharomyces cerevisiae *[1].

Last, we have shown that the variance arising from examining four fold redundant sites, where both transitions and transversions operate, is larger than the variance observed at two fold redundant sites, where only transitions operate. This last point prompted us to directly compare TREx distances with distances obtained from maximum likelihood dS (mldS) calculations, which are now being widely used as a clock when applied to analyses of genome comparison and distant homologs.

Compared to mldS metric, the breadth of the TREx distribution between presumed orthologs is comparable to that of mldS, at least when comparing coding regions of the human, mouse and rat genomes. This is somewhat surprising, as the TREx method uses fewer characters to establish a distance. At the same time, by focusing on a narrower set of data that is presumably "better behaved", the TREx tool discards characters that might cause overdispersion, specifically, the *f*_4 _data that is more overdispersed than the *f*_2 _data. Therefore, the intrinsic dispersion of the TREx clock (that based on the number of characters used to calculate the distance) should always be broader than the dispersion of the mldS clock. Discarding of poorer quality data (that from transversions, here represented by the *f*_4 _data), which leads to a narrower dispersion, balances the effect from discarding data. This means that obtaining the theoretical simplicity of the TREx approach, as well as its shorter computation time (ca. 5 fold faster than mldS) does not require a sacrifice measured in terms of variance.

There is, of course, no reason to believe that the most valuable data to retain, and the selection of data to exclude, will be the same in all lineages over all times. In plants, for example, many of the assumptions that are built into a Poisson model are far worse than in mammals [34]; exclusion of certain characters might be more important. As more genomes are completed, we will be able to assess what data are most useful for constructing clocks for any specific lineage at any specific time. This will allow future research to exploit multiple genomes to estimate the rate constants for transitions and transversions, in multiple contexts, near and far from the chromosomal centromeres, and in the leading and lagging strands (for example), to build a history of transitions and transversion rate constants throughout the history of mammals, and then elsewhere.

Further, as various trees become better articulated, it should be possible to construct ancestral genomes that determine these rate constants throughout the vertebrate lineage. This inference comes from the observation that the TREx sites may not be entirely equilibrated in the time separating fish from mammals. To attain this goal, we will need to proceed stepwise, through the reconstruction of the genome of the last common ancestral placental mammal (using the ancestor of opossum and kangaroo to root the tree), the genome of the last common ancestor of opossum and kangaroo (using the ancestral placental genome to root the tree), the last common ancestor of mammals (using an ancestral avian genome to root), the last common ancestor of the amniotes (using an ancestral fish genome to root the tree), and the last common ancestor of the teleost fish (using the ancestral amniote genome to root the tree), back to the last common ancestor of vertebrates (using the *Ciona *genome to root the tree). The ability to go further back in time through ancestral sequence reconstruction as trees become better articulated has already been demonstrated on synthetic data [46].

## Methods

### Theory

It is well known from chemical kinetics that a two state system interconverting two compounds (here designated by the letters A and G), according to the kinetic scheme:

A⇄kG−−>AkA−−>G G     (1)
 MathType@MTEF@5@5@+=feaafiart1ev1aaatCvAUfKttLearuWrP9MDH5MBPbIqV92AaeXatLxBI9gBaebbnrfifHhDYfgasaacH8akY=wiFfYdH8Gipec8Eeeu0xXdbba9frFj0=OqFfea0dXdd9vqai=hGuQ8kuc9pgc9s8qqaq=dirpe0xb9q8qiLsFr0=vr0=vr0dc8meaabaqaciaacaGaaeqabaqabeGadaaakeaacqqGbbqqdaGdnaWcbaGaem4AaS2aaSbaaWqaaGqaaiab=feabjabgkHiTiabgkHiTiabg6da+iab=DeahbqabaaaleaacqWGRbWAdaWgaaadbaGae83raCKaeyOeI0IaeyOeI0IaeyOpa4dcbiGae4xqaeeabeaaaOGaayPKHiaawcziaiabbccaGiabbEeahjaaxMaacaWLjaWaaeWaaeaacqqGXaqmaiaawIcacaGLPaaaaaa@433E@

approaches equilibrium via an exponential process, where the rate constant *k*_R _is equal to the *sum *of the forward rate constant *and *the reverse rate constant, that is, *k*_R _= *k*_A->G _+ *k*_G->A _[48]. Also well-known is the fact that at equilibrium, the ratio of [G]_eq _to [A]_eq_, where [G]_eq _and [A]_eq _are the respective concentrations of G and A at equilibrium, is equal to the ratio of the forward and reverse rate constants, that is, [G]_eq_/ [A]_eq _= (*k*_A->G_)/(*k*_G->A_). These rate constants can be first order if they reflect a single underlying chemical process. They may appear first order (and hence are called pseudo first order or apparent first order) if they collect many chemical processes that are aggregated into a single rate constant.

This means that if all of the material in a chemical system is A at *t *= 0, where *t *is time, then the fraction of A remaining after time *t*, expressed as *f*_A _= [A(t)]/A_0_, is given by the equation:

[A(t)][A0]=fGeqe−(kA→G+kG→A)t+fAeq     (2)
 MathType@MTEF@5@5@+=feaafiart1ev1aaatCvAUfKttLearuWrP9MDH5MBPbIqV92AaeXatLxBI9gBaebbnrfifHhDYfgasaacH8akY=wiFfYdH8Gipec8Eeeu0xXdbba9frFj0=OqFfea0dXdd9vqai=hGuQ8kuc9pgc9s8qqaq=dirpe0xb9q8qiLsFr0=vr0=vr0dc8meaabaqaciaacaGaaeqabaqabeGadaaakeaadaWcaaqaaiabcUfaBjabdgeabjabcIcaOiabdsha0jabcMcaPiabc2faDbqaaiabcUfaBjabdgeabnaaBaaaleaacqaIWaamaeqaaOGaeiyxa0faaiabg2da9iabdAgaMnaaBaaaleaacqqGhbWrcqqGLbqzcqqGXbqCaeqaaOGaemyzau2aaWbaaSqabeaacqGHsislcqGGOaakcqWGRbWAdaWgaaadbaWexLMBbXgBcf2CPn2qVrwzqf2zLnharyGvLjhzH5wyaGabaiaa=feacqGHsgIRcqqGhbWraeqaaSGaey4kaSIaem4AaS2aaSbaaWqaaiabbEeahjabgkziUkabbgeabbqabaWccqGGPaqkcqWG0baDaaGccqGHRaWkcqWGMbGzdaWgaaWcbaGaeeyqaeKaeeyzauMaeeyCaehabeaakiaaxMaacaWLjaWaaeWaaeaacqaIYaGmaiaawIcacaGLPaaaaaa@646C@

where *f*_Geq _and *f*_Aeq _are the fractions of G and A at equilibrium (that is *f*_Geq _= [G]_eq_/([G]_eq _+ [A]_eq_) and *f*_Aeq _= [A]_eq_/([G]_eq _+ [A]_eq_)).

This expression describes accurately the change in the concentration of A in all time regimes, and captures the process by which an individual A is converted to a G, and then back to A, and then back to G, and so on indefinitely. There is no need to add terms to the equation, or to make corrections to reflect the fact that as the system approaches equilibrium, any particular molecule can undergo an indefinite number of interconversions, back and forth, between the two states. A classic discussion of various correction methods needed in stated-based and event-based models is provided by Gillespie [49].

The fact that corrections are not needed by the formalism presented here can be seen by examining the detailed derivation of Equation (2). We begin by recognizing that the net rate of change in the concentration of A is equal to the rate of conversion of A to G, *minus *the rate of conversion of G back to A. This difference is captured in a differential equation, where each of these microscopic rate processes is equal to the rate constant for the reaction multiplied by the concentration of the reacting species:

−d[A]dt=kA−>G[A]−kG−>A[G]     (3)
 MathType@MTEF@5@5@+=feaafiart1ev1aaatCvAUfKttLearuWrP9MDH5MBPbIqV92AaeXatLxBI9gBaebbnrfifHhDYfgasaacH8akY=wiFfYdH8Gipec8Eeeu0xXdbba9frFj0=OqFfea0dXdd9vqai=hGuQ8kuc9pgc9s8qqaq=dirpe0xb9q8qiLsFr0=vr0=vr0dc8meaabaqaciaacaGaaeqabaqabeGadaaakeaacqGHsisldaWcaaqaaiabdsgaKjabcUfaBjabdgeabjabc2faDbqaaiabdsgaKjabdsha0baacqGH9aqpcqWGRbWAdaWgaaWcbaGaemyqaeKaeyOeI0IaeyOpa4Jaem4raCeabeaakiabcUfaBjabdgeabjabc2faDjabgkHiTiabdUgaRnaaBaaaleaacqWGhbWrcqGHsislcqGH+aGpcqWGbbqqaeqaaOGaei4waSLaem4raCKaeiyxa0LaaCzcaiaaxMaadaqadaqaaiabiodaZaGaayjkaiaawMcaaaaa@4D75@

If the initial concentration of [G] = 0, then at any point during the process, [G] = [A]_0 _- [A]. Substituting this expression for [G] into Equation (3) gives:

−d[A]dt=(kA−>G+kG−>A)[A]−kG−>A[A]0     (4)
 MathType@MTEF@5@5@+=feaafiart1ev1aaatCvAUfKttLearuWrP9MDH5MBPbIqV92AaeXatLxBI9gBaebbnrfifHhDYfgasaacH8akY=wiFfYdH8Gipec8Eeeu0xXdbba9frFj0=OqFfea0dXdd9vqai=hGuQ8kuc9pgc9s8qqaq=dirpe0xb9q8qiLsFr0=vr0=vr0dc8meaabaqaciaacaGaaeqabaqabeGadaaakeaacqGHsisldaWcaaqaaiabdsgaKjabcUfaBjabdgeabjabc2faDbqaaiabdsgaKjabdsha0baacqGH9aqpcqGGOaakcqWGRbWAdaWgaaWcbaGaemyqaeKaeyOeI0IaeyOpa4Jaem4raCeabeaakiabgUcaRiabdUgaRnaaBaaaleaacqWGhbWrcqGHsislcqGH+aGpcqWGbbqqaeqaaOGaeiykaKIaei4waSLaemyqaeKaeiyxa0LaeyOeI0Iaem4AaS2aaSbaaSqaaiabdEeahjabgkHiTiabg6da+iabdgeabbqabaGccqGGBbWwcqWGbbqqcqGGDbqxdaWgaaWcbaGaeGimaadabeaakiaaxMaacaWLjaWaaeWaaeaacqaI0aanaiaawIcacaGLPaaaaaa@56CF@

We then recognize that Equation (4) applies at all points during reaction, including the point when the reaction reaches equilibrium. Letting [A]_eq _represent the equilibrium concentration of A, we write Equation (5), which holds when the reaction reaches equilibrium:

−d[A]eqdt=(kA−>G+kG−>A)[A]eq−kG−>A[A]0     (5)
 MathType@MTEF@5@5@+=feaafiart1ev1aaatCvAUfKttLearuWrP9MDH5MBPbIqV92AaeXatLxBI9gBaebbnrfifHhDYfgasaacH8akY=wiFfYdH8Gipec8Eeeu0xXdbba9frFj0=OqFfea0dXdd9vqai=hGuQ8kuc9pgc9s8qqaq=dirpe0xb9q8qiLsFr0=vr0=vr0dc8meaabaqaciaacaGaaeqabaqabeGadaaakeaacqGHsisldaWcaaqaaiabdsgaKjabcUfaBjabdgeabjabc2faDnaaBaaaleaacqWGLbqzcqWGXbqCaeqaaaGcbaGaemizaqMaemiDaqhaaiabg2da9iabcIcaOiabdUgaRnaaBaaaleaacqWGbbqqcqGHsislcqGH+aGpcqWGhbWraeqaaOGaey4kaSIaem4AaS2aaSbaaSqaaiabdEeahjabgkHiTiabg6da+iabdgeabbqabaGccqGGPaqkcqGGBbWwcqWGbbqqcqGGDbqxdaWgaaWcbaGaemyzauMaemyCaehabeaakiabgkHiTiabdUgaRnaaBaaaleaacqWGhbWrcqGHsislcqGH+aGpcqWGbbqqaeqaaOGaei4waSLaemyqaeKaeiyxa01aaSbaaSqaaiabicdaWaqabaGccaWLjaGaaCzcamaabmaabaGaeGynaudacaGLOaGaayzkaaaaaa@5CB9@

Subtracting Equation (5) from Equation (4) eliminates the k_G->A _[A]_0 _term, giving:

−d([A]−[A]eq)dt=(kA−>G+kG−>A)([A]−[A]eq)=kR([A]−[A]eq)     (6)
 MathType@MTEF@5@5@+=feaafiart1ev1aaatCvAUfKttLearuWrP9MDH5MBPbIqV92AaeXatLxBI9gBaebbnrfifHhDYfgasaacH8akY=wiFfYdH8Gipec8Eeeu0xXdbba9frFj0=OqFfea0dXdd9vqai=hGuQ8kuc9pgc9s8qqaq=dirpe0xb9q8qiLsFr0=vr0=vr0dc8meaabaqaciaacaGaaeqabaqabeGadaaakeaacqGHsisldaWcaaqaaiabdsgaKjabcIcaOiabcUfaBjabdgeabjabc2faDjabgkHiTiabcUfaBjabdgeabjabc2faDnaaBaaaleaacqWGLbqzcqWGXbqCaeqaaOGaeiykaKcabaGaemizaqMaemiDaqhaaiabg2da9iabcIcaOiabdUgaRnaaBaaaleaacqWGbbqqcqGHsislcqGH+aGpcqWGhbWraeqaaOGaey4kaSIaem4AaS2aaSbaaSqaaiabdEeahjabgkHiTiabg6da+iabdgeabbqabaGccqGGPaqkcqGGOaakcqGGBbWwcqWGbbqqcqGGDbqxcqGHsislcqGGBbWwcqWGbbqqcqGGDbqxdaWgaaWcbaGaemyzauMaemyCaehabeaakiabcMcaPiabg2da9iabdUgaRnaaBaaaleaacqWGsbGuaeqaaOGaeiikaGIaei4waSLaemyqaeKaeiyxa0LaeyOeI0Iaei4waSLaemyqaeKaeiyxa01aaSbaaSqaaiabdwgaLjabdghaXbqabaGccqGGPaqkcaWLjaGaaCzcamaabmaabaGaeGOnaydacaGLOaGaayzkaaaaaa@6E38@

This equation is readily integrated in the variable [A] - [A]_eq_, which is the deviation of the concentration of A from the equilibrium value, to give.

ln([*A*]- [*A*]_*eq*_) = -*k*_*R*_*t *    (7)

[A]−[A]eq=e−kRt     (8)
 MathType@MTEF@5@5@+=feaafiart1ev1aaatCvAUfKttLearuWrP9MDH5MBPbIqV92AaeXatLxBI9gBaebbnrfifHhDYfgasaacH8akY=wiFfYdH8Gipec8Eeeu0xXdbba9frFj0=OqFfea0dXdd9vqai=hGuQ8kuc9pgc9s8qqaq=dirpe0xb9q8qiLsFr0=vr0=vr0dc8meaabaqaciaacaGaaeqabaqabeGadaaakeaacqGGBbWwcqWGbbqqcqGGDbqxcqGHsislcqGGBbWwcqWGbbqqcqGGDbqxdaWgaaWcbaGaemyzauMaemyCaehabeaakiabg2da9iabdwgaLnaaCaaaleqabaGaeyOeI0Iaem4AaS2aaSbaaWqaaiabdkfasbqabaWccqWG0baDaaGccaWLjaGaaCzcamaabmaabaGaeGioaGdacaGLOaGaayzkaaaaaa@4320@

[A]=e−kRt+[A]eq     (9)
 MathType@MTEF@5@5@+=feaafiart1ev1aaatCvAUfKttLearuWrP9MDH5MBPbIqV92AaeXatLxBI9gBaebbnrfifHhDYfgasaacH8akY=wiFfYdH8Gipec8Eeeu0xXdbba9frFj0=OqFfea0dXdd9vqai=hGuQ8kuc9pgc9s8qqaq=dirpe0xb9q8qiLsFr0=vr0=vr0dc8meaabaqaciaacaGaaeqabaqabeGadaaakeaacqGGBbWwcqWGbbqqcqGGDbqxcqGH9aqpcqWGLbqzdaahaaWcbeqaaiabgkHiTiabdUgaRnaaBaaameaacqWGsbGuaeqaaSGaemiDaqhaaOGaey4kaSIaei4waSLaemyqaeKaeiyxa01aaSbaaSqaaiabdwgaLjabdghaXbqabaGccaWLjaGaaCzcamaabmaabaGaeGyoaKdacaGLOaGaayzkaaaaaa@4317@

To express this as a fraction of [A_0_], we write:

[A][A0]=e−kRt[A0]+[A]eq[A0]     (10)
 MathType@MTEF@5@5@+=feaafiart1ev1aaatCvAUfKttLearuWrP9MDH5MBPbIqV92AaeXatLxBI9gBaebbnrfifHhDYfgasaacH8akY=wiFfYdH8Gipec8Eeeu0xXdbba9frFj0=OqFfea0dXdd9vqai=hGuQ8kuc9pgc9s8qqaq=dirpe0xb9q8qiLsFr0=vr0=vr0dc8meaabaqaciaacaGaaeqabaqabeGadaaakeaadaWcaaqaaiabcUfaBjabdgeabjabc2faDbqaaiabcUfaBjabdgeabnaaBaaaleaacqaIWaamaeqaaOGaeiyxa0faaiabg2da9maalaaabaGaemyzau2aaWbaaSqabeaacqGHsislcqWGRbWAdaWgaaadbaGaemOuaifabeaaliabdsha0baaaOqaaiabcUfaBjabdgeabnaaBaaaleaacqaIWaamaeqaaOGaeiyxa0faaiabgUcaRmaalaaabaGaei4waSLaemyqaeKaeiyxa01aaSbaaSqaaiabdwgaLjabdghaXbqabaaakeaacqGGBbWwcqWGbbqqdaWgaaWcbaGaeGimaadabeaakiabc2faDbaacaWLjaGaaCzcamaabmaabaGaeGymaeJaeGimaadacaGLOaGaayzkaaaaaa@5232@

This expression for the approach to equilibrium says that in a reversible first-order process, the approach to equilibrium is an apparent first-order kinetic process, with the apparent first order rate constant being (*k*_A->G _+ *k*_G->A_) = *k*_R_. Thus, a plot of ln([A] - [A]_eq_) against *t *will be linear, with slope -*k*_R_, the sum of the forward and reverse rate constants. Again, this equation accurately describes [A] even under conditions where A is transformed back and forth to G an infinite number of times.

This approach-to-equilibrium kinetic model can be applied to the analysis of nucleotide sequence divergence, which is no more (and no less) than a chemical reaction interconverting two chemical states. Here, we adopt (as do others) a null hypothesis that substitution at a site is independent of substitutions at other sites, that the rate constants for substitutions are the same at all sites, and that the ratio at which two species occupy a silent site is the ratio of the forward/reverse rate constants. The last hypothesis simply states that the system is at equilibrium, a good approximation as long as the rate constants are large compared to the rate of change of the rate constants. This constitutes a null hypothesis when examining data.

Consider the case where A and G are nucleotides at *n *sites constrained to accept only purines, because these are the silent, third position, sites of a two-fold redundant codon system for Lys, Glu, or Gln, where the encoded amino acid is conserved throughout the period of evolution being considered. The rate constants *k*_A->G _and *k*_G->A _now correspond to pseudo-first order rate constants for two transition processes at a silent site, the substitution of A by G and the substitution of G by A. Let us assume that these rate constants are time-invariant. We also assume that at *t *= 0, the occupancy of A and G in a site is that expected at equilibrium, *f*_Aeq _and *f*_Geq _respectively, that is, *f*_Geq_/*f*_Aeq _= *f*_G0_/*f*_A0 _= *k*_A->G _/*k*_G->A_, where *f*_G0 _and *f*_A0 _are the fraction of sites at *t *= 0 holding G and A respectively. We also assume that each site suffers mutation independent of other sites, and that the forward and reverse transition rate constants are the same for all sites.

We now consider two identical sequences, where one (note, this is a single lineage rate constant) is given the opportunity to diverge. How will the fraction identity at sites constrained to hold purines diverge in the evolving sequences? Consider separately the sites that are occupied by A at *t *= 0 and the sites that are occupied by G at *t *= 0. For those that are originally occupied by A, the sites conserved after time *t *are those that have A after time *t*.

The conserved sites arising from A is given by Equation 11:

(fGeqe−kRt+fAeq)fAeq     (11)
 MathType@MTEF@5@5@+=feaafiart1ev1aaatCvAUfKttLearuWrP9MDH5MBPbIqV92AaeXatLxBI9gBaebbnrfifHhDYfgasaacH8akY=wiFfYdH8Gipec8Eeeu0xXdbba9frFj0=OqFfea0dXdd9vqai=hGuQ8kuc9pgc9s8qqaq=dirpe0xb9q8qiLsFr0=vr0=vr0dc8meaabaqaciaacaGaaeqabaqabeGadaaakeaacqGGOaakcqWGMbGzdaWgaaWcbaGaee4raCKaeeyzauMaeeyCaehabeaakiabdwgaLnaaCaaaleqabaGaeyOeI0Iaem4AaS2aaSbaaWqaamXvP5wqSXMqHnxAJn0BKvguHDwzZbqegyvzYrwyUfgaiqaacaWFsbaabeaaliabdsha0baakiabgUcaRiabdAgaMnaaBaaaleaacqqGbbqqcqqGLbqzcqqGXbqCaeqaaOGaeiykaKIaemOzay2aaSbaaSqaaiabbgeabjabbwgaLjabbghaXbqabaGccaWLjaGaaCzcamaabmaabaGaeGymaeJaeGymaedacaGLOaGaayzkaaaaaa@54C0@

where the *f*_Aeq _term outside of the parentheses represents the fraction of the starting sites that are occupied by A, while the term within parentheses describes the fraction of these that remain A after time *t*. Note that the parenthetical term is always a number in the range of zero to unity, and that this expression includes the case where A has been converted to G, and then back to A, and so on.

The equation describing the number of conserved sites arising from G as a function of time is similarly derived:

(fAeqe−kRt+fGeq)fGeq     (12)
 MathType@MTEF@5@5@+=feaafiart1ev1aaatCvAUfKttLearuWrP9MDH5MBPbIqV92AaeXatLxBI9gBaebbnrfifHhDYfgasaacH8akY=wiFfYdH8Gipec8Eeeu0xXdbba9frFj0=OqFfea0dXdd9vqai=hGuQ8kuc9pgc9s8qqaq=dirpe0xb9q8qiLsFr0=vr0=vr0dc8meaabaqaciaacaGaaeqabaqabeGadaaakeaacqGGOaakcqWGMbGzdaWgaaWcbaGaeeyqaeKaeeyzauMaeeyCaehabeaakiabdwgaLnaaCaaaleqabaGaeyOeI0Iaem4AaS2aaSbaaWqaaiabbkfasbqabaWccqWG0baDaaGccqGHRaWkcqWGMbGzdaWgaaWcbaGaee4raCKaeeyzauMaeeyCaehabeaakiabcMcaPiabdAgaMnaaBaaaleaacqqGhbWrcqqGLbqzcqqGXbqCaeqaaOGaaCzcaiaaxMaadaqadaqaaiabigdaXiabikdaYaGaayjkaiaawMcaaaaa@4A9B@

The fraction of all sites having the same purine after time *t *as they had at time zero, *f*_2R _is the sum of these two equations:

f2R=fAeq fGeqe−kRt+fAeq fAeq+fAeq fGeqe−kRt+fGeq fGeq     (13)
 MathType@MTEF@5@5@+=feaafiart1ev1aaatCvAUfKttLearuWrP9MDH5MBPbIqV92AaeXatLxBI9gBaebbnrfifHhDYfgasaacH8akY=wiFfYdH8Gipec8Eeeu0xXdbba9frFj0=OqFfea0dXdd9vqai=hGuQ8kuc9pgc9s8qqaq=dirpe0xb9q8qiLsFr0=vr0=vr0dc8meaabaqaciaacaGaaeqabaqabeGadaaakeaacqWGMbGzdaWgaaWcbaGaeGOmaiJaemOuaifabeaakiabg2da9iabdAgaMnaaBaaaleaacqqGbbqqcqqGLbqzcqqGXbqCaeqaaOGaaGPaVlabdAgaMnaaBaaaleaacqqGhbWrcqqGLbqzcqqGXbqCaeqaaOGaemyzau2aaWbaaSqabeaacqGHsislcqWGRbWAdaWgaaadbaGaemOuaifabeaaliabdsha0baakiabgUcaRiabdAgaMnaaBaaaleaacqqGbbqqcqqGLbqzcqqGXbqCaeqaaOGaaGPaVlabdAgaMnaaBaaaleaacqqGbbqqcqqGLbqzcqqGXbqCaeqaaOGaey4kaSIaemOzay2aaSbaaSqaaiabbgeabjabbwgaLjabbghaXbqabaGccaaMc8UaemOzay2aaSbaaSqaaiabbEeahjabbwgaLjabbghaXbqabaGccqWGLbqzdaahaaWcbeqaaiabgkHiTiabdUgaRnaaBaaameaacqqGsbGuaeqaaSGaemiDaqhaaOGaey4kaSIaemOzay2aaSbaaSqaaiabbEeahjabbwgaLjabbghaXbqabaGccaaMc8UaemOzay2aaSbaaSqaaiabbEeahjabbwgaLjabbghaXbqabaGccaWLjaGaaCzcamaabmaabaGaeGymaeJaeG4mamdacaGLOaGaayzkaaaaaa@76D5@

Since *f*_G _+ *f*_A _is always equal to unity, we have :

(*f*_G _+ *f*_A_)^2 ^= 1     (14)

and:

*f*_G _^2 ^+ 2*f*_G_*f*_A _+ *f*_A _^2 ^= 1     (15)

for all *f*_G _and *f*_A_, including *f*_Geq _and *f*_Aeq_. Now, let

*E*_R _= *f*_Geq_^2 ^+ *f*_Aeq_^2 ^    (16)

*P*_R _= 2*f*_Geq_*f*_Aeq_^2 ^    (17)

therefore,

*P*_R _+ *E*_R _= 1     (18)

The equation (13) can therefore be rewritten to give

f2R=PRe−kRt+ER     (19)
 MathType@MTEF@5@5@+=feaafiart1ev1aaatCvAUfKttLearuWrP9MDH5MBPbIqV92AaeXatLxBI9gBaebbnrfifHhDYfgasaacH8akY=wiFfYdH8Gipec8Eeeu0xXdbba9frFj0=OqFfea0dXdd9vqai=hGuQ8kuc9pgc9s8qqaq=dirpe0xb9q8qiLsFr0=vr0=vr0dc8meaabaqaciaacaGaaeqabaqabeGadaaakeaacqWGMbGzdaWgaaWcbaGaeGOmaiJaemOuaifabeaakiabg2da9iabdcfaqnaaBaaaleaaieaacqWFsbGuaeqaaOGaemyzau2aaWbaaSqabeaacqGHsislcqWGRbWAdaWgaaadbaGaeeOuaifabeaaliabdsha0baakiabgUcaRiabdweafnaaBaaaleaacqqGsbGuaeqaaOGaaCzcaiaaxMaadaqadaqaaiabigdaXiabiMda5aGaayjkaiaawMcaaaaa@42AA@

Here, the fraction of conserved purine nucleotides at two fold redundant codon sites follows an exponential first order approach to equilibrium towards an equilibrium end point, *E*_R_, which reflects the equilibrium fractions occupied by A and G. Again, this equation correctly handles the possibility of multiple substitutions at a single site; indeed, this is why the equilibrium is approached.

Solving (19) gives a distance based on transition redundant exchange (TREx) kinetics:

*k*_R_*t*= -ln [*f*_2R _- *E*_R_)/*P*_R_] = TREx distance     (20)

where *P*_R _is the pre-exponential term (= 2*f*_Aeq_*f*_Geq_) and *E*_R _is the *f*_2 _reached at equilibrium (= *f*_Aeq _^2 ^+ *f*_Geq _^2 ^(Fig. 1). A value for *k*_R_*t *can therefore be determined from an *f*_2R _value using Equation (20).

In this model, *f*_2R _as a function of time follows a first order exponential decay from unity to an end point defined by the expression (*f*_Aeq _^2 ^+ *f*_Geq _^2^) (Fig. 1). If A and G appear with equal frequency (for example, if no codon bias exists), then the equilibrium end point *E*_R _= 0.5. If, however, A and G appear with frequencies of (for example) 0.6 and 0.4 in both lineages, then the end point *E*_R _is ca. 0.52 (= 0.6^2 ^+ 0.4^2^).

TREx distance is from ancestor to its descendent and cannot be calculated directly since we do not know the ancestral sequence. To compute the TREx distance between the pairwise aligned sequences, equation (20) is transformed to

*k*_obsR_*t *= -ln [(*f*_2R _- *E*_R_)/*P*_R_] = TREx distance     (21)

where *k*_obsR _is the observed rate constant, which is similar to *k*_R _except that it describes the interconversions between two descendent sequences from the common ancestral sequence instead of between ancestral sequence and its descendent, while *t *is the time from the ancestor to the descendent.

#### Using *f*_2R _and *f*_2Y _to construct molecular clocks

If the rate constants are assumed to be time-invariant, *f*_2R _can be used as a molecular clock. It is a special clock, in that it considers only sites where the amino acid has not diverged, constraining the site to accept only a purine-purine transition. Thus, it exploits only two specific rate constants of the twelve that describe all possible interconversions of the four letters in the genetic alphabet. As discussed below, this formalism becomes especially useful as we estimate those rate constants for ancestral states.

To implement this clock, we identify sites in a pair of aligned DNA sequences that are constrained to mutate between A and G only. The third positions of codons for three amino acids (Glu, Gln, and Lys) are so constrained if the amino acid has not been replaced in the interval separating the two genes. In practice, as non-synonymous substitutions are generally more infrequent than synonymous substitutions, we can ignore the possibility that two compensatory non-synonymous substitutions have led to overall amino acid conservation. We therefore examine a pair of aligned gene sequences for codons encoding a Glu, Gln, and Lys that are conserved between the two encoded proteins, and directly calculate *f*_2R _for the pair of genes by counting identities at the third position sites (*c*) of these codons, and dividing by *n*, the number of such sites.

An analogous kinetic expression can be written for pyrimidine-pyrimidine transitions. The third positions of six amino acids (Cys, Asp, Phe, His, Asn, and Tyr) are constrained to have only T or C, if the encoded amino acid is conserved in the two encoded proteins. Identification in a pair of aligned gene sequences of sites at the third positions of codons that encode these amino acids, where the amino acids are conserved, counting the identities, and dividing by *n*, yields *f*_2Y _(Y for pYrimidines) for the pair of genes. Similar TREx distances can be calculated using a formula analogous to Eq. 21.

### Empirical assessment of the value of the TREx clock

The value of a clock depends on several factors. First, the accuracy of the clock is highest when dating the divergence of genes separated by a time similar to the half-life associated with the transition rate constant, *t*_1/2 _= ln 2/*k*. For events occurring near the time of the divergence of the major mammalian orders ca. 80 million years ago (Ma), for example, the optimal rate constant would be ca. 4.4 × 10^-9 ^transitions/site/year, recognizing that 160 million years in total time separates two contemporary taxa that diverged 80 Ma (note how we have here doubled the time to reflect a double lineage process). Further, the TREx clock would be less valuable if different silent sites within a gene undergo substitution with different rate constants, or different genes undergo silent substitutions with different rate constants. Either of these will create an "overdispersed" clock, where the distribution of *f*_2 _values is larger than expected from a Poisson process given the number of sites used to estimate a distance[50,51]. Last, the clock is less valuable if the rate constants for various transitions are not time-invariant over the period of evolution being considered.

We first assessed the value of the clock by looking for overdispersion in mammals and other vertebrates. To this end, we examined *f*_2R _and *f*_2Y _for a series of inter-taxa pairs of homologous genes for a variety of vertebrate genomes.

For inter-taxon analyses, families in the MasterCatalog (EraGen Biosciences) were identified that contained at least one representative protein from both of the taxa of interest. For these families, all inter-taxa pairs of genes were extracted, together with the pairwise protein sequence alignment. A pairwise alignment of the DNA sequences was then generated to follow the protein sequence alignment. If a family contained more than one sequence of a species belonging to one of the taxa analyzed, then those sequences were checked to determine whether they include redundant sequences (PAM < 1, *f*_2 _> 0.99). If this was the case, only one of the redundant sequences was retained. For *g *genes from one taxon and *h *genes from the other within a family, there were *g*×*h *inter-taxa pairs.

For each pair, the homologous codons that matched identical amino acids in the pairwise protein sequence alignment were then noted, and the identity/non-identity of the nucleotide present at the silent site recorded. Separate statistics were kept for codons of different redundancy (six, four, three, and two fold redundant codon systems). For each pair, the fraction identical, *f*, was recorded for each class of codon, and each type of difference. Thus, *f*_2 _is the fraction of identical nucleotides at two fold redundant sites, *f*_2R _is the fraction of identical nucleotides at two fold redundant sites involving purine-purine transitions, *f*_2Y _is the fraction of identical nucleotides at two fold redundant sites involving pyrimidine-pyrimidine transitions, and *f*_4 _is the fraction of identical nucleotides at four fold redundant sites.

A package was implemented using JAVA, PL/SQL, PERL language and Bioperl toolkit [52]. All computation was carried out in a Class I Beowulf cluster based on an IBM^® ^eServer xSeries 250 (IBM Inc) as a file server, which is facilitated with 4 Intel^® ^Xeon™ processors, 300 GB RAID storage system, and 9 other commodity personal computers (HP Inc) with the installation of Linux operating system (Redhat 8.0). The cluster is networked through a standard 10/100 Mb Ethernet and the data is stored in the file sever using NFS (network file system) protocol. The relational database management systems Mysql and Oracle were used to manipulate the databases.

Sequence manipulations were aided by the Darwin bioinformatics package [53]. The starting point for this analysis was families of all protein sequences contained within GenBank 114. These sequences were extracted, and subjected to an all-against-all comparison [54]. The resulting matches were grouped into a MasterCatalog (EraGen Biosciences, Madison WI) containing 32595 families holding 445185 amino acid sequences. PAM distances between matches were calculated with variances using the Darwin PamEstimator routine. Multiple sequence alignments and evolutionary trees were likewise calculated using the Darwin programming environment. The Darwin package can be obtained by sending an email request to cbrg@inf.ethz.ch.

Codon biases were obtained from the CUTG (Codon Usage Tabulated from GenBank) made available by the Kazusa DNA Research Institute Foundation, Japan (kazusa.or.jp/codon/).

To simulate the expected behaviour within families of proteins, based on the assumption of a random process, a computer program has been developed. As input for the simulation, we require the number of characters. This number, however, differs with different gene pairs. Therefore, as a first step, a Poisson distribution is fit to the number of characters for the *p *protein families using the statistics package presented in Matlab (see Fig. 4 below for an example). From this, the λ value representing the distribution of *n *is determined. Given this λ, a simulation generates the distribution of *f*-values around a mean, based on the null hypothesis that all sites within a gene and all of the gene pairs have diverged with the same rate constants. Thus, if *p *pairs of protein are being used, the characters from each are concatenated to give a supersequence to obtain the midpoint of the distribution. This is then used, with λ, as an input in the simulation to obtain the distribution of *f*-values for the *p *pairs. In the simulation, the Poisson process is assumed, that is, site is equally likely to suffer a substitution, second substitutions are as likely at a site as the first, and the pattern of substitutions is the same at each site.

### Determining variation in f values arising from variation in the rate constants for transitions in different genes

As an approximation (and a null hypothesis), we first assume that all synonymous sites in all two fold redundant codon systems within a gene suffer transitions with the same rate constant. We also assume, when comparing *f *values and TREx distances for different gene pairs, that all genes diverge with the same transition rate constants at synonymous sites. The second approximation is equivalent to the assumption that different genes do not lie in hot and cold spots in the chromosome in the same genome. These assumptions are needed to use TREx distances to order (in rank) dates of divergence of individual paralog pairs within a single genome and to correlate events recorded in the genome with dated events in the paleontological and geological records.

While these approximations certainly generate the simplest model for divergent evolution at synonymous sites, they need not be good to any particular degree of accuracy. It is conceivable that some proteins lie in hot spots on a chromosome. Certain segments of a DNA sequence (CpG islands, for example) are known to undergo change with different rate constants.

For these reasons, the degree to which this approximation holds should be tested empirically. According to the null hypothesis, the expected value for *f *(which represents any type of *f *value, including *f*_2_) should be the same for each pair of homologous proteins diverging at the same time. Because a finite number of characters is used to calculate *f*, the values for *f *should be distributed around this expected value binomially; this converges to a normal distribution when the sample size becomes large. The number of characters used to calculate *f *is typically 125, and the number of genes being compared is typically on the order of several thousand. Thus, the sample size is large enough that a Gaussian curve is a suitable approximation, where the σ value should be a function only of the number of characters used to calculate *f *(in the discussion here, this number is designated *n*). The corresponding Gaussian probability distribution has the following form:

P(f)=1σ2πe−(f0−f)22σ2     (22)
 MathType@MTEF@5@5@+=feaafiart1ev1aaatCvAUfKttLearuWrP9MDH5MBPbIqV92AaeXatLxBI9gBaebbnrfifHhDYfgasaacH8akY=wiFfYdH8Gipec8Eeeu0xXdbba9frFj0=OqFfea0dXdd9vqai=hGuQ8kuc9pgc9s8qqaq=dirpe0xb9q8qiLsFr0=vr0=vr0dc8meaabaqaciaacaGaaeqabaqabeGadaaakeaacqWGqbaucqGGOaakcqWGMbGzcqGGPaqkcqGH9aqpdaWcaaqaaiabigdaXaqaaiabeo8aZnaakaaabaGaeGOmaiJaeqiWdahaleqaaaaakiabdwgaLnaaCaaaleqabaGaeyOeI0YaaSqaaWqaaiabcIcaOiabdAgaMnaaBaaabaGaeGimaadabeaacqGHsislcqWGMbGzcqGGPaqkdaahaaqabeaacqaIYaGmaaaabaGaeGOmaiJaeq4Wdm3aaWbaaeqabaGaeGOmaidaaaaaaaGccaWLjaGaaCzcamaabmaabaGaeGOmaiJaeGOmaidacaGLOaGaayzkaaaaaa@49F3@

where P(*f*) is the probability of a pair having a value of *f*, and *f*_0 _is the mean expectation value for *f*.

If there is also a distribution in the rate constants between different genes, the corresponding expectation values for *f *should also be distributed, however. This implies that the observed distribution in the values of *f *should be broader than the theoretical distribution arising from a finite *n*. This is because a variance in underlying rate constants will create a breadth in the *f *distribution, as well as the fact that *f *is calculated from a finite set of characters.

No good arguments exist to choose a particular distribution for the expected *f*_0 _values. We have therefore simply assumed that the rate constants are distributed log normally, creating a distribution of the expectation values for *f *for different genes that is distributed normally around *f*_0_. In other words:

D(fk)=1ρ2πe−(fk0−fk)22ρ2     (23)
 MathType@MTEF@5@5@+=feaafiart1ev1aaatCvAUfKttLearuWrP9MDH5MBPbIqV92AaeXatLxBI9gBaebbnrfifHhDYfgasaacH8akY=wiFfYdH8Gipec8Eeeu0xXdbba9frFj0=OqFfea0dXdd9vqai=hGuQ8kuc9pgc9s8qqaq=dirpe0xb9q8qiLsFr0=vr0=vr0dc8meaabaqaciaacaGaaeqabaqabeGadaaakeaacqWGebarcqGGOaakcqWGMbGzdaWgaaWcbaGaem4AaSgabeaakiabcMcaPiabg2da9maalaaabaGaeGymaedabaGaeqyWdi3aaOaaaeaacqaIYaGmcqaHapaCaSqabaaaaOGaemyzau2aaWbaaSqabeaacqGHsisldaWcbaadbaGaeiikaGIaemOzay2aaSbaaeaacqWGRbWAcqaIWaamaeqaaiabgkHiTiabdAgaMnaaBaaabaGaem4AaSgabeaacqGGPaqkdaahaaqabeaacqaIYaGmaaaabaGaeGOmaiJaeqyWdi3aaWbaaeqabaGaeGOmaidaaaaaaaGccaWLjaGaaCzcamaabmaabaGaeGOmaiJaeG4mamdacaGLOaGaayzkaaaaaa@4E4B@

where D(*f*_*k*_) is the distribution of genes with different expectation values for *f *(*f*_*k*_), centered on *f*_*k*0_, where ρ representing the standard deviation for the distribution.

These two distributions can be convoluted to create a new distribution using to the following integral:

N(f)=12πρσ∫−∞+∞e−(fk0−fk)22ρ2e−(fk−f)22σ2dfk     (24)
 MathType@MTEF@5@5@+=feaafiart1ev1aaatCvAUfKttLearuWrP9MDH5MBPbIqV92AaeXatLxBI9gBaebbnrfifHhDYfgasaacH8akY=wiFfYdH8Gipec8Eeeu0xXdbba9frFj0=OqFfea0dXdd9vqai=hGuQ8kuc9pgc9s8qqaq=dirpe0xb9q8qiLsFr0=vr0=vr0dc8meaabaqaciaacaGaaeqabaqabeGadaaakeaacqWGobGtcqGGOaakcqWGMbGzcqGGPaqkcqGH9aqpdaWcaaqaaiabigdaXaqaaiabikdaYiabec8aWjabeg8aYjabeo8aZbaadaWdXaqaaiabdwgaLnaaCaaaleqabaGaeyOeI0YaaSqaaWqaaiabcIcaOiabdAgaMnaaBaaabaGaem4AaSMaeGimaadabeaacqGHsislcqWGMbGzdaWgaaqaaiabdUgaRbqabaGaeiykaKYaaWbaaeqabaGaeGOmaidaaaqaaiabikdaYiabeg8aYnaaCaaabeqaaiabikdaYaaaaaaaaaWcbaGaeyOeI0IaeyOhIukabaGaey4kaSIaeyOhIukaniabgUIiYdGccqWGLbqzdaahaaWcbeqaaiabgkHiTmaaleaameaacqGGOaakcqWGMbGzdaWgaaqaaiabdUgaRbqabaGaeyOeI0IaemOzayMaeiykaKYaaWbaaeqabaGaeGOmaidaaaqaaiabikdaYiabeo8aZnaaCaaabeqaaiabikdaYaaaaaaaaOGaemizaqMaemOzay2aaSbaaSqaaiabdUgaRbqabaGccaWLjaGaaCzcamaabmaabaGaeGOmaiJaeGinaqdacaGLOaGaayzkaaaaaa@67B9@

Solving this integral using Maple, followed by normalization to ensure that the definite integral (over the range -infinity to infinity) is equal to unity,

N(f)=12π(ρ2+σ2)e−(k0−f)22(ρ2+σ2)     (25)
 MathType@MTEF@5@5@+=feaafiart1ev1aaatCvAUfKttLearuWrP9MDH5MBPbIqV92AaeXatLxBI9gBaebbnrfifHhDYfgasaacH8akY=wiFfYdH8Gipec8Eeeu0xXdbba9frFj0=OqFfea0dXdd9vqai=hGuQ8kuc9pgc9s8qqaq=dirpe0xb9q8qiLsFr0=vr0=vr0dc8meaabaqaciaacaGaaeqabaqabeGadaaakeaacqWGobGtcqGGOaakcqWGMbGzcqGGPaqkcqGH9aqpdaWcaaqaaiabigdaXaqaamaakaaabaGaeGOmaiJaeqiWdaNaeiikaGIaeqyWdi3aaWbaaSqabeaacqaIYaGmaaGccqGHRaWkcqaHdpWCdaahaaWcbeqaaiabikdaYaaakiabcMcaPaWcbeaaaaGccqWGLbqzdaahaaWcbeqaaiabgkHiTmaaleaameaacqGGOaakcqWGRbWAdaWgaaqaaiabicdaWaqabaGaeyOeI0IaemOzayMaeiykaKYaaWbaaeqabaGaeGOmaidaaaqaaiabikdaYiabcIcaOiabeg8aYnaaCaaabeqaaiabikdaYaaacqGHRaWkcqaHdpWCdaahaaqabeaacqaIYaGmaaGaeiykaKcaaaaakiaaxMaacaWLjaWaaeWaaeaacqaIYaGmcqaI1aqnaiaawIcacaGLPaaaaaa@560D@

This expression is in the form of a normal distribution, where the apparent standard deviation σ_app _is related to the theoretical σ and ρ by the expression:

σapp=ρ2+σ2     (26)
 MathType@MTEF@5@5@+=feaafiart1ev1aaatCvAUfKttLearuWrP9MDH5MBPbIqV92AaeXatLxBI9gBaebbnrfifHhDYfgasaacH8akY=wiFfYdH8Gipec8Eeeu0xXdbba9frFj0=OqFfea0dXdd9vqai=hGuQ8kuc9pgc9s8qqaq=dirpe0xb9q8qiLsFr0=vr0=vr0dc8meaabaqaciaacaGaaeqabaqabeGadaaakeaacqaHdpWCdaWgaaWcbaGaemyyaeMaemiCaaNaemiCaahabeaakiabg2da9maakaaabaGaeqyWdi3aaWbaaSqabeaacqaIYaGmaaGccqGHRaWkcqaHdpWCdaahaaWcbeqaaiabikdaYaaaaeqaaOGaaCzcaiaaxMaadaqadaqaaiabikdaYiabiAda2aGaayjkaiaawMcaaaaa@3F48@

This relationship allows us to estimate the breadth of the observed distribution in *f *values that arises from different genes in a collection having different rate constants, if the number of characters used to calculate the distribution *n *is known. First, one determines the value for σ expected for the collection based on the value of *n*. Then, one fits a normal distribution to the set of experimental data showing a distribution of *f *values, and estimates a value for σ_app_. One then obtains a value for ρ from equation (12), obtained from equation (11).

ρ=σapp2−σ2     (27)
 MathType@MTEF@5@5@+=feaafiart1ev1aaatCvAUfKttLearuWrP9MDH5MBPbIqV92AaeXatLxBI9gBaebbnrfifHhDYfgasaacH8akY=wiFfYdH8Gipec8Eeeu0xXdbba9frFj0=OqFfea0dXdd9vqai=hGuQ8kuc9pgc9s8qqaq=dirpe0xb9q8qiLsFr0=vr0=vr0dc8meaabaqaciaacaGaaeqabaqabeGadaaakeaacqaHbpGCcqGH9aqpdaGcaaqaaiabeo8aZnaaDaaaleaacqWGHbqycqWGWbaCcqWGWbaCaeaacqaIYaGmaaGccqGHsislcqaHdpWCdaahaaWcbeqaaiabikdaYaaaaeqaaOGaaCzcaiaaxMaadaqadaqaaiabikdaYiabiEda3aGaayjkaiaawMcaaaaa@3F1F@

This provides an estimate of the distribution in the expectation values for *f *that rise from different genes in the set diverging with different transition rate constants.

## Authors' contributions

TL developed TREx technology, implemented the related software packages, performed statistics analysis and helped to prepare the manuscript; SGC provided MasterCatalog, tested whether the overdispersion observed in various *f*_2 _values correlated with dispersity, and analyzed orthologs from curated databases; MDC and DL began the study; EAG helped to prepare the manuscript; SAB proposed the TREx technology, performed TREx analysis and prepared the manuscript.
